# Early coronary revascularization among ‘stable’ patients with non-ST-segment elevation acute coronary syndromes: the role of diabetes and age

**DOI:** 10.1093/cvr/cvae190

**Published:** 2024-08-28

**Authors:** Natalia Fabin, Edina Cenko, Maria Bergami, Jinsung Yoon, Giuseppe Vadalà, Guiomar Mendieta, Sasko Kedev, Jorgo Kostov, Marija Vavlukis, Elif Vraynko, Davor Miličić, Zorana Vasiljevic, Marija Zdravkovic, Lina Badimon, Alfredo R Galassi, Olivia Manfrini, Raffaele Bugiardini

**Affiliations:** Laboratory of Epidemiological and Clinical Cardiology, Department of Medical and Surgical Sciences, University of Bologna, Policlinico Sant’Orsola Malpighi, Padiglione 11, Via Massarenti 9, 40138 Bologna, Italy; Laboratory of Epidemiological and Clinical Cardiology, Department of Medical and Surgical Sciences, University of Bologna, Policlinico Sant’Orsola Malpighi, Padiglione 11, Via Massarenti 9, 40138 Bologna, Italy; Laboratory of Epidemiological and Clinical Cardiology, Department of Medical and Surgical Sciences, University of Bologna, Policlinico Sant’Orsola Malpighi, Padiglione 11, Via Massarenti 9, 40138 Bologna, Italy; Google Cloud Space, AI Department, Sunnyvale, CA, USA; Division of Cardiology, University Hospital Paolo Giaccone, Palermo, Italy; Servicio de Cardiología, Institut Clínic Cardiovascular, Hospital Clínic de Barcelona, Barcelona, Spain; Hospital Clinic de Barcelona, Institut d'Investigacions Biomèdiques August Pi i Sunyer (IDIBAPS), Barcelona, Spain; Centro Nacional de Investigaciones Cardiovasculares Carlos III (CNIC), Madrid, Spain; Sts. Cyril and Methodius University, University Clinic for Cardiology, Skopje, Republic of North Macedonia; Faculty of Medicine, Ss. Cyril and Methodius University in Skopje, 1000 Skopje, Republic of North Macedonia; Sts. Cyril and Methodius University, University Clinic for Cardiology, Skopje, Republic of North Macedonia; Faculty of Medicine, Ss. Cyril and Methodius University in Skopje, 1000 Skopje, Republic of North Macedonia; Sts. Cyril and Methodius University, University Clinic for Cardiology, Skopje, Republic of North Macedonia; Faculty of Medicine, Ss. Cyril and Methodius University in Skopje, 1000 Skopje, Republic of North Macedonia; Sts. Cyril and Methodius University, University Clinic for Cardiology, Skopje, Republic of North Macedonia; Department for Cardiovascular Diseases, University Hospital Center Zagreb, University of Zagreb, Zagreb, Croatia; Medical Faculty, University of Belgrade, Belgrade, Serbia; Faculty of Medicine, University of Belgrade, Clinical Hospital Center Bezanijska kosa, Belgrade, Serbia; Cardiovascular Program-ICCC, Institut d'Investigació Biomèdica Sant Pau (IIB SANT PAU), 08041 Barcelona, Spain; Centro de Investigación Biomédica en Red Cardiovascular (CIBERCV), Instituto de Salud Carlos III, 28029 Madrid, Spain; Cardiovascular Research Chair, Universitat Autònoma de Barcelona (UAB), 08193 Barcelona, Spain; Department of Health Promotion, Mother and Child Care, Internal Medicine and Medical Specialties (ProMISE), University of Palermo, Palermo, Italy; Laboratory of Epidemiological and Clinical Cardiology, Department of Medical and Surgical Sciences, University of Bologna, Policlinico Sant’Orsola Malpighi, Padiglione 11, Via Massarenti 9, 40138 Bologna, Italy; IRCCS Azienda Ospedaliero-Universitaria di Bologna, Sant'Orsola Hospital, Bologna, Italy; Laboratory of Epidemiological and Clinical Cardiology, Department of Medical and Surgical Sciences, University of Bologna, Policlinico Sant’Orsola Malpighi, Padiglione 11, Via Massarenti 9, 40138 Bologna, Italy

**Keywords:** NSTE-ACS, Diabetes, Revascularization, Risk stratification

## Abstract

**Aims:**

To investigate the impact of an early coronary revascularization (<24 h) compared with initial conservative strategy on clinical outcomes in diabetic patients with non-ST-segment elevation acute coronary syndrome (NSTE-ACS) who are in stable condition at hospital admission.

**Methods and results:**

The International Survey of Acute Coronary Syndromes database was queried for a sample of diabetic and nondiabetic patients with diagnosis of NSTE-ACS. Patients with cardiac arrest, haemodynamic instability, and serious ventricular arrhythmias were excluded. The characteristics between groups were adjusted using logistic regression and inverse probability of treatment weighting models. Primary outcome measure was all-cause 30-day mortality. Risk ratios (RRs) and odds ratios (ORs) with their 95% confidence intervals (CIs) were employed. Of the 7589 NSTE-ACS patients identified, 2343 were diabetics. The data show a notable reduction in mortality for the elderly (>65 years) undergoing early revascularization compared to those receiving an initial conservative strategy both in the diabetic (3.3% vs. 6.7%; RR: 0.48; 95% CI: 0.28–0.80) and nondiabetic patients (2.7% vs. 4.7%: RR: 0.57; 95% CI: 0.36–0.90). In multivariate analyses, diabetes was a strong independent predictor of mortality in the elderly (OR: 1.43; 95% CI: 1.03–1.99), but not in the younger patients (OR: 1.04; 95% CI: 0.53–2.06).

**Conclusion:**

Early coronary revascularization does not lead to any survival advantage within 30 days from admission in young NSTE-ACS patients who present to hospital in stable conditions with and without diabetes. An early invasive management strategy may be best reserved for the elderly. Factors beyond revascularization are of considerable importance for outcome in elderly diabetic subjects with NSTE-ACS.

**Clinical trial number:**

ClinicalTrials.gov: NCT01218776.

## Graphical Abstract

**Graphical Abstract:**
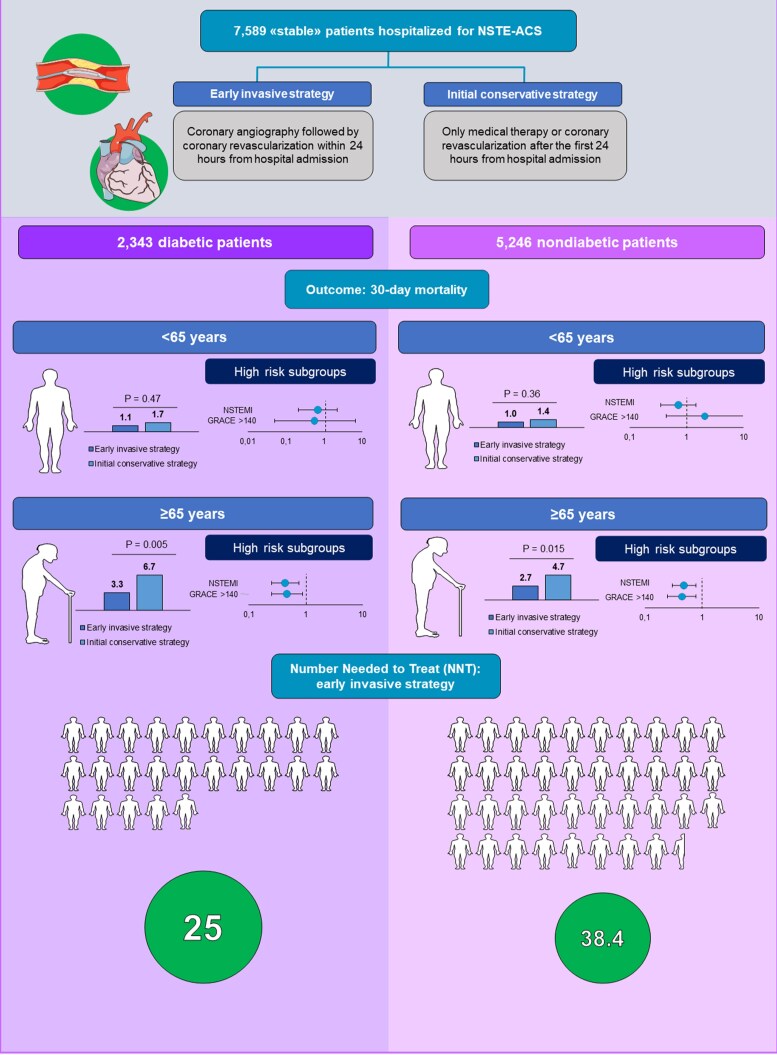

**Time of primary review: 102 days**

## 1. Introduction

Diabetes mellitus is common among patients hospitalized with non-ST-segment elevation acute coronary syndrome (NSTE-ACS), with a reported prevalence of between 10 and 30%.^1–4^ Mortality of NSTE-ACS patients with diabetes can be twice as high as for those without diabetes.^5–7^ Moreover, patients with diabetes and NSTE-ACS have an increased risk of complications following percutaneous coronary intervention (PCI) compared with nondiabetic patients.^8^

Despite the high rate of post PCI complications in diabetes, contemporary guidelines from the European Society of Cardiology (ESC)^9^ suggest to proceed with an early, within 24 h, invasive coronary strategy irrespective of diabetic status in all patients with confirmed non-ST-segment elevation myocardial infarction (NSTEMI). As well, the recently published American College of Cardiology Foundation/American Heart Association (ACCF/AHA)^10^ on myocardial revascularization align in their recommendations to proceed with an early strategy irrespective of diabetic status in patients considered to be of high risk of clinical events defined as those with a Global Registry of Acute Coronary Events (GRACE) score of ≥140.

These recommendations, however, are mainly based on experts’ opinion, and little, if any, published work exists that examines the impact of an early, within 24 h, revascularization on clinical outcomes in patients whose condition can safely be stabilized in the coronary care unit.^11^ ‘Stable’ in this context means that patients are not in immediate critical conditions, although they still require medical attention. Issues that might influence outcomes and pose specific problems include diabetes and age, which may both impact the severity of coronary artery disease (CAD), myocardial function, and the overall risk profile of patients.^12,13^

The current study aims to investigate whether NSTE-ACS patients who are in stable conditions at hospital admission benefit more from early, within 24 h, revascularization compared with an initial conservative strategy considering the complexities that diabetes and age can introduce into the clinical course of the disease. This information could inform better clinical decision-making using the principles of benefit-based tailored treatment.

## 2. Methods

### 2.1 Study design and setting

The International Survey of Acute Coronary Syndromes (ISACS-TC; clinicaltrials.gov: NCT01218776) is a large, prospective, multicentre cohort study. Details of the study design, sampling, and recruitment have been previously published.^14–18^ Adhesion to the current analysis was given by eight collaborating centres from seven European countries: Bosnia and Herzegovina, Croatia, Italy, Macedonia, Montenegro, Romania, and Serbia. All these centres were tertiary health care services providing PCI and cardiac surgery. This study complies with the Declaration of Helsinki.^19^ The data-coordinating centre has been established at the University of Bologna. The local research Ethics Committee from each hospital approved the study. Because patient information was collected anonymously, institutional review boards waived the need for individual informed consent. All data were transferred to the Department of Electrical and Computer Engineering, University of California, Los Angeles, where final statistical analyses were done.

### 2.2 Study population

The designated physician collected the registry data at the time of clinical assessment. All eligible patients must have presented to the hospital with chest pain not occurring >24 h prior to admission. In addition to chest pain, patients must have documented ST-segment depression on the electrocardiogram (ECG) and/or evidence of myocardial necrosis (troponin concentration >99th centile using sex-specific upper reference limit on presentation or subsequent testing).^20^ An early invasive strategy was defined as coronary angiography with or without revascularization, either PCI or coronary artery bypass graft (CABG), with procedure time being within 24 h of admission. The remaining patients were defined as an initial conservative strategy group. This definition of an early invasive strategy in NSTE-ACS has previously been used for other large observational studies.^11,21,22^ The selection of the mode of revascularization (PCI or CABG) was based on patients’ characteristics and preferences. Information regarding diabetic status was extracted from medical charts and information supplied by the patient. All patients categorized as diabetic were on current antidiabetic medications. Information on diabetes was collected blinded to the outcomes.

### 2.3 Eligibility criteria

To meet eligibility criteria, patients had to be admitted in clinically stable conditions. We, therefore, applied the following exclusion criteria: life-threatening arrhythmias or cardiac arrest after presentation, cardiogenic shock (Killip Class 4), acute severe heart failure (Killip Class 3). These criteria would have suggested immediate urgent revascularization being the favoured therapeutic approach as opposed to initial conservative strategy.^23,24^ To avoid immortal time bias—as patients who were selected for the study would have to survive enough to have the procedure—a landmark analysis was used. We defined the landmark time as 24 h from time of hospitalization. The analysis evaluated patient outcomes from the landmark time through to the end of the follow-up period, censored at 30 days from date of hospitalization.

### 2.4 Patient selection on the intention-to-treat principle and efficacy of revascularization

There were patients undergoing angiography within 24 h who did not receive revascularization. This suggests that no significant lesion was found, or that revascularization was deemed unnecessary or inappropriate. Including these patients in the early invasive strategy group would be a logical decision, based on the intention-to-treat principle. However, some considerations should be done. The primary benefit of revascularization in NSTE-ACS is typically observed in patients with significant coronary lesions. Including patients who underwent angiography within 24 h but did not receive revascularization could potentially dilute the potential benefits. This is because their risk profile and outcomes could be substantially different from those who required revascularization. This concern addresses a key principle in clinical research: ensuring that the study population accurately reflects the intervention being evaluated. To circumvent this issue, we conducted the primary analyses both with and without these patients. This approach allows for a clearer interpretation of the data enabling us to compare how the inclusion of these patients affects the efficacy of the early invasive strategy.

### 2.5 Outcomes

Primary outcome measure of the study was all-cause 30-day mortality. The 30-day window for mortality was selected to enrich the data over that acquired during the index hospitalization while mitigating survivor bias. Other outcomes of interest were length of stay, major bleeding, and PCI complications. Major bleeding was defined as a decrease in blood haemoglobin level of at least 5 g/dL, the occurrence of intracranial haemorrhage or cardiac tamponade, fatal bleeding, or any combination of these events.^25^ PCI complications that may have had significant impact on patient survival were rare. As such, they were combined in a single variable including no reflow (Thrombolysis in Myocardial Infarction 0–2) grading system,^26^ coronary perforation or dissection, acute coronary thrombosis, coronary artery side branch loss, distal embolization, and elevated troponin post PCI intervention.^20^ We did not include recurrence of symptoms in our outcome measures as in most of the previous trials, recurrent ischaemic events were driven by ‘symptoms of ischaemia’ but what this entails is uncertain and, therefore, is a soft endpoint at risk of bias.^27^

### 2.6 Concomitant therapies and definitions

We also noted the type of evidence-based medications given on hospital admission and during hospitalization until discharge. Medical therapy on admission included aspirin and P2Y_12_ inhibitors. Other standard treatments were given during hospitalization including angiotensin-converting enzyme inhibitors (ACE-inhibitors), angiotensin receptor blockers (ARBs), beta-blockers, and statins. However, information on timing of in-hospital medications’ initiation was not systematically available in the database. As such, analyses on their effects on outcomes were not evaluated due to the possible persistence of immortal time bias. Smoking habits were self-reported. Hypertension and hypercholesterolaemia were assessed by documentation of medical history prior to admission in the database (see Supplementary material online, Methods). The GRACE risk score was calculated for each patient.^28^ All patients with a glomerular filtration rate <60 mL/min/1.73  m^2^ for 3 months were defined as having chronic kidney disease.^29^ Based on the coronary arteriographic findings, multivessel disease was defined as at least two main branches of the epicardial coronary artery with ≥70% stenotic lesions or ≥50% stenosis in the left main coronary artery.^30^

### 2.7 Statistical analysis

We compared the baseline characteristics and clinical outcomes of patients who received an initial conservative strategy with those who received an early invasive strategy. Analyses were stratified by age (<65 or ≥65 years) and diabetic status. Other exploratory analyses included the criteria indicative of increased risk: NSTEMI and GRACE risk score of >140. Baseline characteristics were reported as number (percentages) for categorical variables and mean ± standard deviation for continuous variables. Statistical testing was performed with the use of Pearson’s *χ*^2^ test for categorical variables and the two-sample *t*-test for continuous variables. A two-sided *P* value of <0.05 was considered statistically significant. Each patient record detailed 23 clinical features and 8 medications (see Supplementary material online, *Table S1*). We used inverse probability of treatment weighting (IPTW) based on the propensity score for confounding adjustment (see Supplementary material online, Methods). To reduce the imbalance of potential confounding factors between the two treatment strategies, we compiled a set of baseline covariates as listed in Supplementary material online, *Table S1*. Variables included in the models were demographic, cardiovascular risk factors, comorbidities (history of ischaemic heart disease, cardiovascular disease, and other comorbidities, namely chronic kidney disease), and clinical features on hospital presentation. The occurrence of other possible interactions between the invasive strategy and other factors was evaluated by logistic multiple regression analysis. We had complete data on diabetes status and 30-day mortality. Among the variables included in the IPTW models, missingness was not considerable (<30%)^31^ (see Supplementary material online, *Table S2*). We used Multiple Imputation with Chained Equations as imputation method to treat missing data^32^ (see Supplementary material online, Methods). We reported the coefficient estimates, clustered adjust standard errors, *T* statistics, and corresponding *P* values in Supplementary material online, *Table S3*. Standardized differences after weighting were calculated to ensure balanced treatment groups with respect to baseline characteristics. Groups were considered balanced when the standardized difference was <10% (see Supplementary material online, Methods). Risk ratios (RRs) and odds ratios (ORs) with their 95% confidence intervals (CIs) were employed (see Supplementary material online, Methods). Comparisons of outcomes between groups were made by two-sided *P* value of <0.05 (see Supplementary material online, Methods). All statistical analyses were performed using R, version 4.2.1 (R Foundation for Statistical Computing, Vienna, Austria).

## 3. Results

A total of 9069 with NSTE-ACS were enrolled from the ISACS-TC participating hospitals between October 2010 and July 2023. From this group, 258 patients were excluded because they had evidence of cardiogenic shock (Killip Class 4) or acute heart failure (Killip Class 3) on hospital presentation. In addition, 135 patients were excluded because they died (*n* = 67) or had life-threatening arrhythmias or cardiac arrest (*n* = 68) before the landmark time. Moreover, 938 patients were excluded as angiography was not followed by revascularization in the first 24 h (*n* = 580) or they had incomplete data on the timing of angiography (*n* = 358). Lastly, 149 patients were excluded because they had missing data concerning their diabetes status. The final cohort consisted of 7589 patients. Of these, 2343 were diabetic patients while 5246 were nondiabetics (see Supplementary material online, *Figure S1*). We included the 580 patients who underwent angiography within 24 h, but did not receive revascularization in the sensitivity analyses shown below. This inclusion may provide a more comprehensive view of the early invasive strategy outcomes.

### 3.1 Baseline characteristics of the overall study population stratified by treatment strategy

The baseline characteristics of the 7589 NSTE-ACS patients stratified by treatment strategy are presented in Supplementary material online, *Table S1*. A total of 3513 patients (46.3%) underwent an early invasive strategy during their admission. Of the 4076 patients who were treated with initial conservative strategy, 39.4% underwent later revascularization within the 30-day study. The timing of revascularization of these patients is shown in Supplementary material online, *Figure S2*. An early invasive strategy was associated with a reduction in length of stay [median duration: 6 days (4–9) vs. 4 days (3–6), *P* < 0.001] (see Supplementary material online, *Figure S3*).

### 3.2 Baseline characteristics of patients with and without diabetes stratified by treatment strategy

Among the overall study population, we identified 2343 diabetic patients (mean age, 67.1 ± 10.2 years; 38.2% women) (*Table 1*) and 5246 nondiabetic patients (mean age, 63.3 ± 12.0 years; 29.6% women) (*Table 2*). A lower proportion of patients with diabetes than those without underwent an early invasive strategy (43.4% vs. 47.6%). Baseline differences between treatment strategy groups were similar in patients with and without diabetes. Compared with an initial conservative strategy, patients undergoing an early invasive strategy were significantly (standardized difference ≥10%) younger, more often male, and more likely to be admitted to a cardiology service with a diagnosis of NSTEMI. Patients who received an early invasive strategy had lower unadjusted rates for 30-day mortality.

**Table 1:** Baseline characteristics of the NSTE-ACS diabetic population stratified by treatment strategy

Characteristics	Diabetic patients				
	Overall population (*n* = 2343)	Early invasive strategy (*n* = 1016)	Initial conservative strategy (*n* = 1327)	*P* value^a^	Standardized mean difference^a^
Mean age (SD), years	67.1 (10.2)	65.7 (9.9)	68.1 (10.4)	<0.001	−0.24
Women	896 (38.2)	352 (34.6)	544 (41.8)	0.001	−0.13
Cardiovascular risk factors					
Hypercholesterolaemia	1244 (53.1)	556 (54.7)	688 (51.8)	0.16	0.05
Hypertension	2031 (86.7)	881 (86.7)	1150 (86.7)	0.97	0.001
Current smokers	570 (24.3)	299 (29.4)	271 (20.4)	<0.001	0.20
Family history of CAD	803 (34.3)	362 (35.6)	441 (33.2)	0.22	0.05
History of ischaemic heart disease					
Chronic coronary syndrome	788 (33.6)	309 (30.4)	479 (36.1)	0.003	−0.12
Prior myocardial infarction	653 (27.9)	266 (26.2)	387 (29.2)	0.10	−0.06
Prior CABG	153 (6.5)	49 (4.8)	104 (7.8)	0.002	−0.12
Prior PCI	443 (18.9)	233 (22.9)	210 (15.8)	<0.001	0.18
History of cardiovascular disease					
Peripheral artery disease	111 (4.7)	39 (3.8)	72 (5.4)	0.06	−0.07
Prior heart failure	215 (9.2)	78 (7.7)	137 (10.3)	0.02	−0.09
Prior stroke or TIA	137 (5.8)	46 (4.5)	91 (6.9)	0.01	−0.10
Other comorbidities					
Chronic kidney disease	339 (14.5)	135 (13.3)	204 (15.4)	0.15	−0.05
Clinical presentation on hospital admission					
Mean heart rate (SD), b.p.m.	84.6 (20.2)	81.1 (18.3)	87.3 (21.2)	<0.001	−0.31
Mean SBP (SD), mmHg	143.8 (26.6)	146.3 (25.4)	141.8 (27.4)	<0.001	0.17
NSTEMI	1848 (78.9)	832 (81.9)	1016 (76.6)	0.001	0.13
UA	480 (20.5)	184 (18.1)	296 (22.3)	0.01	−0.10
Medications taken before hospitalization					
Antiplatelet medications	1299 (55.4)	572 (56.3)	727 (54.8)	0.46	0.03
ACE-inhibitors/ARBs	1522 (65.0)	636 (62.6)	886 (66.8)	0.03	−0.08
Beta-blockers	1276 (54.5)	573 (56.4)	703 (53.0)	0.09	0.06
Statins	1145 (48.9)	495 (48.7)	650 (49.0)	0.89	−0.005
Medications administered on hospital admission					
Antiplatelet medications	2306 (98.4)	1011 (99.5)	1295 (97.6)	<0.001	0.16
Medications administered during hospitalization and at discharge					
Beta-blockers	1871 (79.9)	786 (77.4)	1085 (81.8)	0.009	−0.10
ACE-inhibitors/ARBs	1972 (84.2)	855 (84.2)	1117 (84.2)	0.98	−0.0006
Statins	2190 (93.5)	969 (95.4)	1221 (92.0)	<0.001	0.13
Revascularization type					
PCI	1351 (57.7)	996 (98.0)	355 (26.8)	<0.001	2.17
CABG	245 (10.5)	105 (10.3)	140 (10.6)	0.86	−0.007
In-hospital complications					
Major bleeding	52 (2.2)	9 (0.9)	43 (3.2)	<0.001	−0.16
PCI complications	77 (3.3)	47 (4.6)	30 (2.3)	0.002	0.13
Outcomes				*P* value	
30-day mortality	99 (4.2)	24 (2.4)	75 (5.7)	<0.001	
Risk ratio (95% CI)		0.40 (0.25–0.64)		<0.001	

ACE, angiotensin-converting enzyme; ARBs, angiotensin receptor blockers; b.p.m., beats per minute; CABG, coronary artery bypass graft; CAD, coronary artery disease; NSTE-ACS, non-ST-segment elevation acute coronary syndromes; NSTEMI, non-ST-segment elevation myocardial infarction; PCI, percutaneous coronary intervention; SBP, systolic blood pressure; TIA, transient ischaemic attack; UA, unstable angina.

^a^Calculated between early invasive strategy and initial conservative strategy groups.

**Table 2:** Baseline characteristics of the NSTE-ACS nondiabetic patients stratified by treatment strategy

Characteristics	Nondiabetic patients				
	Overall population (*n* = 5246)	Early invasive strategy (*n* = 2497)	Initial conservative strategy (*n* = 2749)	*P* value^a^	Standardized mean difference^a^
Mean age (SD), years	63.3 (12.0)	62.2 (11.2)	64.3 (12.6)	<0.001	−0.17
Women	1554 (29.6)	643 (25.8)	911 (33.1)	<0.001	−0.16
Cardiovascular risk factors					
Hypercholesterolaemia	2435 (46.4)	1217 (48.7)	1218 (44.3)	0.001	0.08
Hypertension	3869 (73.8)	1798 (72.0)	2071 (75.3)	0.006	−0.07
Current smokers	1916 (36.5)	1008 (40.4)	908 (33.0)	<0.001	0.15
Family history of CAD	1844 (35.2)	900 (36.0)	944 (34.3)	0.19	0.03
History of ischaemic heart disease					
Chronic coronary syndrome	1513 (28.8)	625 (25.0)	888 (32.3)	<0.001	−0.16
Prior myocardial infarction	1109 (21.1)	499 (20.0)	610 (22.2)	0.05	−0.05
Prior CABG	173 (3.3)	48 (1.9)	125 (4.5)	<0.001	−0.14
Prior PCI	744 (14.2)	402 (16.1)	342 (12.4)	<0.001	0.10
History of cardiovascular disease					
Peripheral artery disease	170 (3.2)	92 (3.7)	78 (2.8)	0.08	0.04
Prior heart failure	247 (4.7)	81 (3.2)	166 (6.0)	<0.001	−0.13
Prior stroke or TIA	236 (4.5)	85 (3.4)	151 (5.5)	<0.001	−0.10
Other comorbidities					
Chronic kidney disease	335 (6.4)	139 (5.6)	196 (7.1)	0.02	−0.06
Clinical presentation on hospital admission					
Mean heart rate (SD), b.p.m.	81.1 (19.7)	79.2 (18.2)	82.9 (2.8)	<0.001	−0.18
Mean SBP (SD), mmHg	143.1 (25.5)	143.4 (25.6)	142.9 (25.3)	0.43	0.02
NSTEMI	4079 (77.8)	2044 (81.9)	2035 (74.0)	<0.001	0.18
UA	1167 (22.2)	453 (18.1)	714 (26.0)	<0.001	−0.18
Medications taken before hospitalization					
Antiplatelet medications	2249 (42.9)	1056 (42.3)	1193 (43.4)	0.41	−0.02
ACE-inhibitors/ARBs	2553 (48.7)	1188 (47.6)	1365 (49.7)	0.13	−0.04
Beta-blockers	2132 (40.6)	988 (39.6)	1144 (41.6)	0.13	−0.04
Statins	1597 (30.4)	817 (32.7)	780 (28.4)	<0.001	0.09
Medications administered on hospital admission					
Antiplatelet medications	5157 (98.3)	2483 (99.4)	2674 (97.3)	<0.001	0.17
Medications administered during hospitalization and at discharge					
Beta-blockers	4031 (76.8)	1875 (75.1)	2156 (78.4)	0.004	−0.07
ACE-inhibitors/ARBs	4053 (77.3)	1933 (77.4)	2120 (77.1)	0.79	0.007
Statins	4923 (93.8)	2412 (96.6)	2511 (91.3)	<0.001	0.22
Revascularization type					
PCI	3338 (63.6)	2463 (98.6)	875 (31.8)	<0.001	1.93
CABG	443 (8.4)	176 (7.0)	267 (9.7)	<0.001	−0.09
In-hospital complications					
Major bleeding	60 (1.1)	26 (1.0)	34 (1.2)	0.50	−0.01
PCI complications	109 (2.1)	83 (3.3)	26 (0.9)	<0.001	0.16
Outcomes				*P* value	
30-day mortality	126 (2.4)	38 (1.5)	88 (3.2)	<0.001	
Risk ratio (95% CI)		0.47 (0.32–0.69)		<0.001	

ACE, angiotensin-converting enzyme; ARBs, angiotensin receptor blockers; b.p.m., beats per minute; CABG, coronary artery bypass graft; CAD, coronary artery disease; NSTE-ACS, non-ST-segment elevation acute coronary syndromes; NSTEMI, non-ST-segment elevation myocardial infarction; PCI, percutaneous coronary intervention; SBP, systolic blood pressure; TIA, transient ischaemic attack; UA, unstable angina.

^a^Calculated between early invasive strategy and initial conservative strategy groups.

### 3.3 Care patterns

Patients who underwent early invasive management were statistically more likely to receive antiplatelet agents on hospital admission compared with patients who underwent early conservative management in both the diabetic and nondiabetic population (*Tables 1* and *2*). Treatment instituted during hospitalization, as revealed by ongoing therapy at hospital discharge, differed between the two groups of management. Patients who underwent early revascularization were more frequently given beta-blockers and statins in both the diabetic and nondiabetic population (*Tables 1* and *2*).

### 3.4 Angiographic findings

Coronary angiography was available for 3942 (75.1%) of the nondiabetic and for 1653 (70.6%) of the diabetic patients, respectively. Supplementary material online, *Figure S4*, convincingly demonstrates the significantly more widespread CAD among the diabetic cohort, with as many as 43.0% of these patients categorized as having multivessel CAD compared with 34.2% of the nondiabetic patients (*P* < 0.001).

### 3.5 Inverse probability-of-treatment weighting models stratified by diabetes status

Diabetic patients in the early invasive and conservative groups were well balanced after IPTW with standardized difference < 10% for all covariates (*Table 3*). The rate of death at 30 days in the weighted sample was 2.5% for the early invasive strategy group and 4.8% for the initial conservative strategy group (RR: 0.50; 95% CI: 0.31–0.80). Comparable patterns of outcomes were observed in the nondiabetic population (*Table 3*).

**Table 3:** IPTW: clinical factors and outcomes stratified by treatment strategy and diabetes status

Characteristics	Diabetic patients			Nondiabetic patients		
	Early invasive strategy (*n* = 1016)	Initial conservative strategy (*n* = 1327)	Standardized mean difference	Early invasive strategy (*n* = 2497)	Initial conservative strategy (*n* = 2749)	Standardized mean difference
Mean age (SD), years	66.9 (9.8)	66.9 (10.6)	−0.006	63.1 (11.2)	63.2 (12.5)	−0.003
Women	38.7	38.6	0.002	29.5	29.5	−0.0001
Cardiovascular risk factors						
Hypercholesterolaemia	53.2	53.1	0.003	46.2	46.3	−0.0006
Hypertension	86.9	86.7	0.005	73.7	73.7	−0.0003
Current smoking	24.6	24.8	−0.003	36.8	36.7	0.0003
Family history of CAD	34.1	34.1	0.0001	35.0	34.9	0.001
History of ischaemic heart disease						
Chronic coronary syndrome	33.4	33.2	0.003	28.5	28.8	−0.006
Prior myocardial infarction	26.4	27.2	−0.01	21.2	21.2	0.0007
Prior CABG	6.3	6.4	−0.005	3.3	3.3	0.002
Prior PCI	18.8	18.6	0.004	14.3	14.2	0.001
History of cardiovascular disease						
Peripheral artery disease	4.5	4.7	−0.01	3.0	3.0	0.0005
Prior heart failure	9.3	9.2	0.003	4.7	4.7	−0.0006
Prior stroke or TIA	6.0	5.9	0.06	4.8	4.6	0.009
Other comorbidities						
Chronic kidney disease	14.0	14.3	−0.008	6.6	6.3	0.01
Clinical presentation on hospital admission						
Mean heart rate (SD), b.p.m.	85.3 (22.8)	84.6 (19.8)	0.03	81.3 (20.2)	81.2 (19.7)	0.004
Mean SBP (SD), mmHg	143.8 (26.2)	143.9 (27.0)	−0.002	142.9 (26.1)	142. (24.9)	−0.004
Outcomes			*P* value			*P* value
30-day mortality	2.5	4.8	0.003	1.9	2.9	0.01
Risk ratio (95% CI)	0.50 (0.31–0.80)		0.003	0.62 (0.43–0.90)		0.01

Data are expressed as weighted means (standard deviation) or weighted percentages, unless otherwise specified.

b.p.m., beats per minute; CABG, coronary artery bypass graft; CAD, coronary artery disease; PCI, percutaneous coronary intervention; SBP, systolic blood pressure; TIA, transient ischaemic attack.

### 3.6 Inverse probability-of-treatment weighting models stratified by age and diabetes status

Stratification by age gave a different perspective of risk. Risk reduction with an early invasive strategy was consistent among the elderly in the diabetic population (65 years and older) with an absolute difference in death of 3.4% (RR: 0.48; 95% CI: 0.28–0.80) (*Table 4*). In contrast, younger age had no significant association with death (absolute difference: 0.6%; RR: 0.66; 95% CI: 0.21–2.06) (*Table 4*). Similar results were observed in the nondiabetic population (*Table 5*).

**Table 4:** IPTW: clinical factors and outcomes stratified by age subgroups and treatment strategy in diabetic patients

Characteristics	Diabetic patients					
	Age <65 years			Age ≥65 years		
	Early invasive strategy (*n* = 428)	Initial conservative strategy (*n* = 471)	Standardized mean difference	Early invasive strategy (*n* = 588)	Initial conservative strategy (*n* = 856)	Standardized mean difference
Mean age (SD), years	56.5 (5.9)	56.5 (6.7)	0.008	73.4 (5.5)	73.5 (6.4)	−0.01
Women	28.9	29.3	−0.009	44.8	44.5	0.006
Cardiovascular risk factors						
Hypercholesterolemia	53.2	53.3	−0.001	53.1	52.8	0.004
Hypertension	80.4	80.0	0.01	90.9	90.9	−0.0002
Current smoking	38.7	38.9	−0.004	15.7	15.9	−0.007
Family history of CAD	41.8	40.9	0.01	29.0	29.6	−0.01
History of ischaemic heart disease						
Chronic coronary syndrome	32.1	31.5	0.01	34.6	34.2	0.007
Prior myocardial infarction	25.6	24.8	0.01	27.1	28.7	−0.03
Prior CABG	6.3	5.6	0.02	6.5	7.0	−0.01
Prior PCI	18.2	17.3	0.02	19.0	19.2	−0.007
History of cardiovascular disease						
Peripheral artery disease	3.4	3.6	−0.009	5.1	5.4	−0.01
Prior heart failure	7.1	7.4	−0.01	10.6	10.2	0.01
Prior stroke or TIA	4.2	4.2	0.0005	7.4	6.9	0.01
Other comorbidities						
Chronic kidney disease	8.2	7.0	0.04	17.7	18.7	−0.02
Clinical presentation on hospital admission						
Mean heart rate (SD), b.p.m.	85.9 (23.9)	85.6 (18.7)	0.03	84.9 (22.2)	84.5 (20.4)	0.02
Mean SBP (SD), mmHg	144.9 (26.9)	145.1 (29.3)	−0.007	142.9 (25.7)	143.0 (26.7)	−0.003
Outcomes			*P* value			*P* value
30-day mortality	1.1	1.7	0.47	3.3	6.7	0.005
Risk ratio (95% CI)	0.66 (0.21–2.06)		0.47	0.48 (0.28–0.80)		0.005

Data are expressed as weighted means (standard deviation) or weighted percentages, unless otherwise specified.

b.p.m., beats per minute; CABG, coronary artery bypass graft; CAD, coronary artery disease; PCI, percutaneous coronary intervention; SBP, systolic blood pressure; TIA, transient ischaemic attack.

**Table 5:** IPTW: clinical factors and outcomes stratified by age subgroups and treatment strategy in nondiabetic patients

Characteristics	Nondiabetic patients					
	Age <65 years			Age ≥65 years		
	Early invasive strategy (*n* = 1449)	Initial conservative strategy (*n* = 1443)	Standardized mean difference	Early invasive strategy (*n* = 1048)	Initial conservative strategy (*n* = 1306)	Standardized mean difference
Mean age (SD), years	54.4 (7.2)	54.4 (7.3)	0.002	74.0 (6.2)	74.1 (6.4)	−0.01
Women	23.9	23.9	−0.0003	36.5	36.3	0.003
Cardiovascular risk factors						
Hypercholesterolaemia	47.0	46.9	0.001	45.6	45.0	0.01
Hypertension	66.8	66.6	0.004	82.9	82.8	0.004
Current smoking	50.7	50.6	0.001	19.5	19.6	−0.002
Family history of CAD	38.2	38.3	−0.001	30.3	30.2	0.001
History of ischaemic heart disease						
Chronic coronary syndrome	24.8	25.0	0.005	33.3	33.7	−0.007
Prior myocardial infarction	19.5	19.7	−0.004	23.0	22.5	0.01
Prior CABG	2.0	2.0	0.001	5.2	5.0	0.01
Prior PCI	14.4	14.3	0.002	14.4	14.2	0.005
History of cardiovascular disease						
Peripheral artery disease	1.7	1.6	0.004	4.6	4.7	−0.004
Prior heart failure	2.5	2.6	−0.004	7.4	7.4	−0.002
Prior stroke or TIA	2.9	2.8	0.006	7.0	6.6	0.01
Other comorbidities						
Chronic kidney disease	3.3	3.2	0.005	10.7	10.3	0.01
Clinical presentation on hospital admission						
Mean heart rate (SD), b.p.m.	80.4 (18.7)	80.4 (18.4)	−0.0007	82.0 (21.4)	82.1 (21.3)	−0.002
Mean SBP (SD), mmHg	143.7 (25.9)	143.6 (24.6)	0.004	141.8 (26.2)	142.1 (25.4)	−0.01
Outcomes			*P* value			*P* value
30-day mortality	1.0	1.4	0.36	2.7	4.7	0.01
Risk ratio (95% CI)	0.73 (0.37–1.44)		0.36	0.57 (0.36–0.90)		0.01

Data are expressed as weighted means (standard deviation) or weighted percentages, unless otherwise specified.

b.p.m., beats per minute; CABG, coronary artery bypass graft; CAD, coronary artery disease; PCI, percutaneous coronary intervention; SBP, systolic blood pressure; TIA, transient ischaemic attack.

### 3.7 Age and subgroup analyses based on high baseline risk

**Figure 1:**
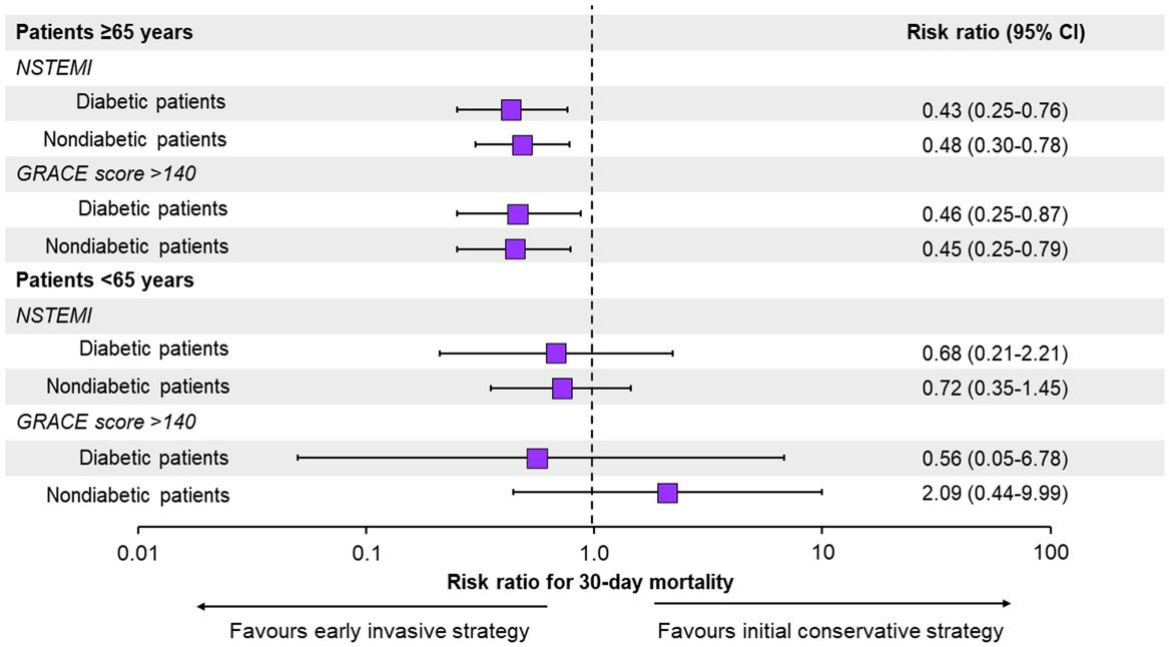
Subgroup analyses of patients with NSTEMI and GRACE risk score >140 stratified by age category and diabetes status. RRs and 95% CI obtained through IPTW analyses. A base-10 log scale is used for the *X* axis. GRACE, Global Registry of Acute Coronary Events; NSTEMI, non-ST-segment elevation myocardial infarction. Population size: elderly patients (≥65 years), diabetic = 737, elderly patients (≥65 years), nondiabetic = 1149, younger patients (<65 years), diabetic = 114, younger patients (<65 years), nondiabetic = 198.

Compared with the primary analyses, there were no substantial changes in the patterns of the primary outcomes in subgroups stratified according to age and the presence of NSTEMI or GRACE risk score >140. In the elderly population, the incidence of death was lower with an early invasive strategy either in the diabetic or nondiabetic population (*Figure 1*, Supplementary material online, *Tables S4–S7*). In contrast, younger age had no significant association with the outcome of death in the presence of NSTEMI or GRACE risk score >140 (*Figure 1*, Supplementary material online, *Tables S8–S11*).

### 3.8 Subgroup reanalysis using RRs between the two intervention groups

**Figure 2:**
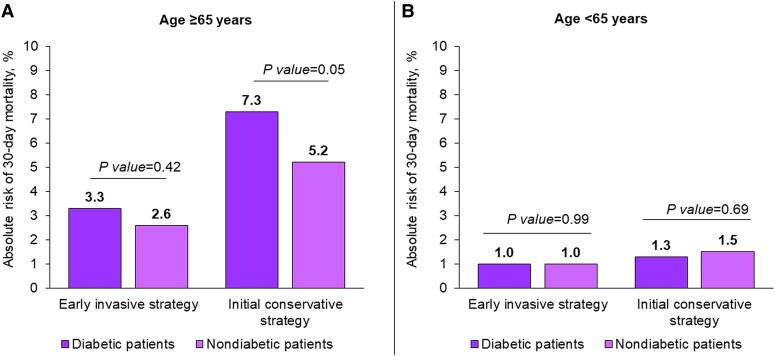
Absolute risk of 30-day mortality in the elderly patients (Panel A) and younger patients (Panel B) stratified by treatment strategy. 30-day mortality rates obtained through IPTW analyses. Population size: elderly patients (≥65 years) undergoing early invasive strategy, diabetic = 588; elderly patients (≥65 years) undergoing initial conservative strategy, diabetic = 856; younger patients (<65 years) undergoing early invasive strategy, diabetic = 428; younger patients (<65 years) undergoing initial conservative strategy, diabetic = 471; elderly patients (≥65 years) undergoing early invasive strategy, nondiabetic = 1048; elderly patients (≥65 years) undergoing initial conservative strategy, nondiabetic = 1306; younger patients (<65 years) undergoing early invasive strategy, nondiabetic = 1449; younger patients (<65 years) undergoing initial conservative strategy, nondiabetic = 1443.

We compared outcome data between the two subgroups of patients (diabetic and nondiabetic patients) for each type of intervention (early invasive or initial conservative strategy). Unlike the standard approach, this methodology incorporates all covariate balancing conditions between the subgroup population undergoing each therapeutic strategy (*Figure 2A*, Supplementary material online, *Tables S12* and *S13*). Among the older population, the impact of an early invasive strategy was approximately of the same magnitude in both diabetic and nondiabetic patients. The occurrence of death in diabetic and nondiabetic patients was 3.3 and 2.6%, respectively (RR: 1.27; 95% CI: 0.70–2.30). The corresponding event rates with an initial conservative management were higher in both groups, but with a larger relative as well as absolute risk in diabetic compared with nondiabetic patients (7.3% vs. 5.2%; RR: 1.42; 95% CI: 1.00–2.03). Outcomes did not differ between diabetic and nondiabetic patients in the younger population (*Figure 2B*, Supplementary material online, *Tables S14* and *S15*). In the context of the elderly population, the absolute risk reduction with an early invasive strategy in the diabetic patients was 4% (7.3–3.3%) compared with 2.6% (5.2–2.6%) in their counterparts. The corresponding number needed to treat was 25 (100:4) for diabetic people and 38.4 (100:2.6) for nondiabetic people.

### 3.9 Safety outcomes

Among patients who underwent PCI, diabetic patients had a higher weighted rate of periprocedural PCI complications compared with nondiabetic patients (4.5% vs. 3.2%, RR: 1.43; 95% CI 1.03–1.96) (see Supplementary material online, *Figure S5*A and B and *Tables S16–S18*). Periprocedural PCI complications were similar between the early intervention group and the conservative strategy group both in diabetics (4.8% vs. 6.0%, RR: 0.78; 95% CI: 0.46–1.33) and nondiabetic patients (3.4% vs. 3.0%, RR: 1.14; 95% CI: 0.73–1.78). The weighted rate of major bleeding (see Supplementary material online, *Figure S5C* and *D* and *Tables S19*–*S21*) was higher in diabetic compared with nondiabetic patients (2.1% vs. 1.1%, RR: 1.90, 95% CI: 1.30–2.78). Yet, patients in the early invasive strategy had a significantly reduced rate of major bleeding both in diabetic (0.8% vs. 2.8%, RR: 0.26; 95% CI: 0.12–0.57) and nondiabetic patients (1.0% vs. 1.2%, RR: 0.86; 95% CI: 0.51–1.44).

### 3.10 Multivariable analyses

**Figure 3:**
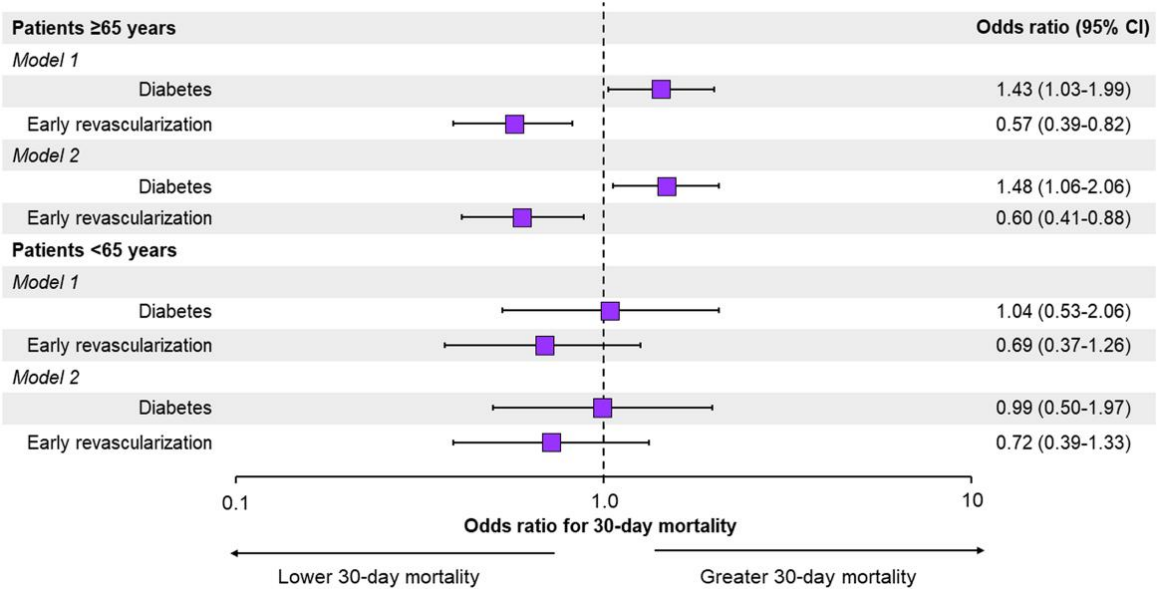
Multivariable logistic regression models for 30-day mortality. Model 1: adjusted for demographic characteristics, cardiovascular risk factors, history of ischaemic heart disease, history of cardiovascular disease, other comorbidities, clinical presentation on hospital admission, and early revascularization. Model 2: Model 1 + antiplatelet medications. A base-10 log scale is used for the *X* axis. Population size: 7589 patients (entire cohort).

Because diabetic patients are a population at higher risk than nondiabetic patients, multivariate statistics were applied to investigate whether diabetes as such was an independent risk predictor in both the elderly and the younger patients. Diabetes was a strong independent predictor of death in the elderly (OR: 1.43; 95% CI: 1.03–1.99), but not in the younger patients (OR: 1.04; 95% CI: 0.53–2.06), which underlines the interplay between diabetes and age for the outcome (*Figure 3*). To reinforce our data, we also estimated the multivariable-adjusted effect of early revascularization on 30-day mortality in function of age. The ORs for death with early revascularization were 0.57 (95% CI: 0.39–0.82) in the elderly and 0.69 (95% CI: 0.37–1.26) in the younger patients. These associations did not differ according to treatment with antiplatelet medications (*Figure 3*). It follows that logistic regression adjustment resulted in conclusions similar to those obtained using IPTW methods.

### 3.11 Data analysed by the intention-to-treat principle

We assessed the stability and reliability of our primary results by including in the analyses the 580 patients who underwent early angiography without subsequent revascularization. We observed a similar pattern of results with the inclusion of these additional patients. The risk reduction with an early invasive strategy among the elderly diabetic with an absolute difference in death of 2.9% (RR: 0.55; 95% CI: 0.34–0.89) indicates a substantial benefit in this subgroup (see Supplementary material online, *Table S22*). In contrast, younger age had no significant association with death (absolute difference: 0.7%; RR: 0.63; 95% CI: 0.23–1.76). Similar results were observed in the nondiabetic population (see Supplementary material online, *Table S23*).

## 4. Discussion

This study provides insights on real-life management strategies of patients with NSTE-ACS who are in stable condition at hospital admission. The principal finding of the current analysis is that a strategy of routine early coronary revascularization was associated with an increased 30-day survival only among patients aged 65 years and older. Although an early invasive strategy in the elderly was of benefit in both diabetic and nondiabetic patients, the effect in people with diabetes was substantially larger than in the nondiabetic subjects. Approximately 25 patients with diabetes had to be treated for one patient to survive with an invasive strategy, compared with 38 nondiabetic patients. This information implies that the early invasive strategy had a higher impact among elderly patients with diabetes compared to those without diabetes.

Although only exploratory, these findings raise strong concern about the use of early coronary revascularization in all patients who have stabilized after a NSTE-ACS, even in those with diabetes.

### 4.1 Prior work on timing of intervention in NSTE-ACS

Within the field of clinical practice, it is common knowledge that patients with NSTE-ACS presenting with recurrent chest pain, haemodynamic instability or cardiogenic shock, acute heart failure, and life-threatening arrhythmias or cardiac arrest may benefit from early within 2-h coronary revascularization.^10,23^ As such, these categories of patients were excluded from our study. In contrast, it remains uncertain whether patients whose condition can safely be stabilized in the coronary care unit should routinely receive an initial, within 24 h, invasive strategy. In this stable population, current guidelines recommend an early strategy for all patients with NSTEMI.^9^ They also advise an early invasive strategy in patients with a GRACE risk score >140 or with dynamic ECG changes suggesting ongoing ischaemia. The scientific base supporting the <24-h invasive guideline recommendation is primarily provided by two subgroup analyses of the Timing of Intervention in Acute Coronary Syndromes (TIMACS)^33^ and Very Early Versus Deferred Invasive Evaluation Using Computerized Tomography (VERDICT) trials.^34^ However, these analyses combined low- and high-risk patients, including those with haemodynamic instability or prior cardiac arrest who were not clearly in a stable phase of their disease. Moreover, they found no significant difference in all-cause mortality even among the subgroup of patients considered to be at the highest risk. Previous meta-analyses^27,35,36^ also found no significant difference in hard clinical endpoints between early and delayed invasive strategies in NSTE-ACS. Thus, the survival benefit of early coronary revascularization remains unclear, especially for patients who met stabilization criteria. These patients were the focus of our investigation. In these patients, the prognosis is uncertain, and the predictive value of age and diabetes has not yet been ascertained.

### 4.2 Prior work on diabetic patients

No randomized trials have compared early revascularization with conservative management in diabetic patients. A meta-analysis of nine randomized trials examined the benefit of an invasive strategy in diabetic patients with NSTE-ACS. This meta-analysis found more nonfatal myocardial infarctions over 12 months in those not receiving routine revascularization, but no outcome at earlier time points was specified.^37^ Another meta-analysis of eight trials suggested an early invasive strategy might reduce mortality at 180 days in high-risk patients, including those with elevated biomarkers, diabetes, or aged 75 years and older.^36^ However, most studies predate 2010. Thus, prior work offers limited information on contemporary treatments and related outcomes of patients with combination of NSTE-ACS and diabetes.

### 4.3 Early coronary revascularization and heterogeneity of treatment effect

The results of the IPTW analyses indicated that diabetic and nondiabetic patients undergoing an early, within 24 h, invasive coronary strategy had odds of death at 30 days that were significantly lower than the odds among their counterparts treated with initial conservative strategy. However, these data estimate an average treatment effect that implicitly assumes a similar treatment effect across heterogeneous patient characteristics, and patients with NSTE-ACS are a very heterogeneous population. As such, the treatment effect in some subgroups of patients may vary considerably from the average effect.^38^ In line with these thoughts, we investigated if the observed treatment difference was the same for young and old patients and for diabetic and nondiabetic patients. Exploratory analyses on this issue were reasonable as there was a specific prior suspicion of the existence of age- and diabetes-based differences in the pathophysiology and outcomes of NSTE-ACS.^39–43^

### 4.4 Treatment effect modification by age

In the current study, elderly patients (aged ≥ 65 years) seemed to benefit most from an early invasive approach. There was an absolute difference of 3.4% in the rate of death from any cause between an early invasive and an early conservative strategy in the elderly diabetic population. In contrast, early coronary revascularization was not associated with a significant decreased risk of death among young diabetic patients. Similar patterns were observed in the nondiabetic population. Therefore, these data suggest that most patients with NSTE-ACS do not need to be rushed to the catheterization laboratory if they are in stable conditions. On this background, an early invasive management strategy may be best reserved for elderly patients irrespective of the diabetes status.

### 4.5 Treatment effect modification by age and diabetes

Elderly with diabetes had a significantly higher rate of death than did nondiabetic patients both in the early invasive and conservative strategies. However, the relative impact of an early invasive strategy was of greater magnitude in diabetic than nondiabetic patients. The absolute risk reduction of death with the early invasive strategy was 4% (from 7.3 to 3.3%) in the diabetic patients compared with 2.6% (from 5.2 to 2.6%) in their counterparts without diabetes. Another way of expressing this disparity is the number needed to treat. If 4 diabetic patients out of 100 benefit more from the early invasive strategy, the number needed to treat to save one life is about 25 patients. The corresponding number in the nondiabetic population is 38 patients. These data, therefore, suggest that early revascularization may mitigate the negative impact of diabetes on elderly NSTE-ACS patients’ outcomes.

### 4.6 Patients categorized as high risk by guidelines

The survival benefit of an early invasive strategy in the elderly was also demonstrated among a range of predefined high-risk subgroups such as patients with NSTEMI or GRACE risk score > 140. These findings were comparable for both diabetic and nondiabetic patients. In line with the primary analyses, our results did not show superiority of an invasive strategy over a conservative approach in the younger population of patients. Our findings, therefore, imply that the decision of when to perform revascularization in patients with NSTE-ACS who are in stable condition at hospital admission should not solely be based on whether the patient has NSTEMI or a high GRACE risk score. A more personalized approach should be taken, considering additional factors. These factors might include the patient’s overall health status and coexisting medical conditions which, in turn, can often be summarized by age. On the other hand, it cannot be ignored that the GRACE risk score combines several clinical variables, such as heart rate, systolic blood pressure, serum creatinine, and Killip classes all measuring different aspects of the same underlying pathophysiologic phenomenon, specifically acute haemodynamic instability, whereas the focus of our study was on patients who were in a stable phase of their disease.

### 4.7 Mechanisms of interplay between diabetes and age for cardiovascular outcomes

Notably, the data we have provided suggests that diabetes is a strong independent predictor of death in the elderly (OR: 1.43; 95% CI: 1.03–1.99), but not in the younger population (OR: 1.04; 95% CI: 0.53–2.06). This finding underscores the interplay between age and diabetes in influencing outcomes among NSTE-ACS patients. Older adults are generally more susceptible to various health conditions due to the natural aging process and the accumulation of health-related issues over time. Functional changes in older hearts include increased oxidative stress, inflammation, apoptosis and overall myocardial deterioration, and degeneration, which may trigger left ventricular dysfunction.^40^ Diabetes is associated with chronic inflammation, endothelial dysfunction, and metabolic abnormalities.^41^ As such, diabetic patients tend to have much more diffuse microcirculatory disease, poorer myocardial perfusion, and more risk of left ventricular dysfunction.^42^ In summary, diabetes can act as an amplifier of age-related health issues, making its impact more pronounced in the elderly population. In contrast, younger patients might have better physiological resilience and other factors such as obesity that may influence the mortality risk more than diabetes.^43^

### 4.8 Safety and cost-efficacy outcomes

While acknowledging the benefits of early invasive strategies in the elderly, and even more in the elderly with diabetes, it is essential to rigorously consider the safety and cost-effectiveness of such an approach. Our study’s finding that there is no excess risk for major bleeding and periprocedural PCI complications associated with an early invasive strategy is reassuring from a safety perspective. The observation of a significantly shorter length of hospital stay for patients undergoing an early invasive strategy may also have positive implications for cost-effectiveness, as shorter hospitalizations generally reduce healthcare costs. Data on the length of hospital stay are concordant with those of previous metanalyses.^27,35,36^ Yet, there are limited studies providing comprehensive insights into the economic implications of this approach.^44^

### 4.9 Limitations

Our study should be interpreted in the context of several potential limitations. First, this analysis is not a randomized study. Although the propensity-based IPTW helps to adjust for differences between groups, it does not control for unmeasured differences in clinical care. However, as a randomized trial cannot be carried out for every subgroup of patients, an observational database is helpful in providing hypothesis-generating data on the heterogeneity of treatment effects. Secondly, treatment algorithms might have changed over time between 2010 and 2023. Over a period of 13 years, advancements in the diagnostic and treatment modalities for NSTE-ACS have evolved significantly. The present results were obtained with limited use of second-generation ultrathin strut drug-eluting stents (DESs). However, ultrathin strut DESs may not be suitable for a variety of lesion subsets largely represented in the diabetic population such as heavily calcified lesions, ostial lesions, and chronic total occlusions. Importantly, ∼20% of the included patients were biomarker negative using conventional troponin assays and thus they could be classified as unstable angina. The proportion of the patients labelled as ‘unstable angina’ may be in fact greater using contemporary high-sensitivity troponin assays. This could have diluted any potential treatment effect from an early invasive strategy in patients defined as NSTEMI in the current analysis. However, the balance in the distribution of unstable angina patients across treatment groups helps to mitigate concerns about potential biases in the observed outcomes. Thirdly, the present study did not define whether an early intervention should be a PCI or a CABG. This decision was at the discretion of the physicians. It is therefore not possible, on the basis of our data, to elaborate on the choice of revascularization procedure for the diabetic compared with the nondiabetic patients. Fourthly, the limited duration of follow-up may obscure the possibility of later survival benefit. Finally, subgroup analyses can only be considered hypothesis generating as mentioned above.

## 5. Conclusions

The research question is relevant, as an early invasive strategy in all patients with NSTE-ACS is a logistical challenge, which requires hospitals with PCI availability and, therefore, important changes in the network hospital organization. There has been continued debate over the last 10 years whether ‘immediate’ or ‘early’ angiography and revascularization is beneficial compared with a more ‘early conservative’ approach. None of the prior studies, however, have tried to address this topic among patients whose condition can safely be stabilized after NSTE-ACS. In our study, we observed a strong and robust heterogeneity in the treatment effects of an initial invasive strategy in clinically stable NSTE-ACS patients. This indicates that a one-size-fits-all approach may not be appropriate for NSTE-ACS management. Our data suggests that not all patients with NSTE-ACS need to be rushed to the catheterization laboratory if they are in stable conditions. Prioritizing patients who are most likely to benefit from early revascularization can optimize resource utilization without compromising patient care. Elderly patients benefit more from early revascularization. Old diabetic patients may have even greater benefit with an early intervention. This complexity suggests that patient-specific factors, such as age and diabetes, must be carefully considered in clinical decision-making. Future randomized controlled trials would provide more robust evidence to confirm or refine our findings.

## Supplementary Material

cvae190_Supplementary_Data

## Data Availability

To guarantee the confidentiality of personal and health information, only the authors have had access to the data during the study. The source codes for this manuscript are uploaded on GitHub (https://github.com/jsyoon0823/Treatment_Phenotype).
