# Supplementary material for: Early coronary revascularization among ‘stable’ patients with non-ST-segment elevation acute coronary syndromes: the role of diabetes and age
Source: Cardiovasc Res. 2024 Aug 28;120(16):2064–77. doi: 10.1093/cvr/cvae190 (PMC12709607; doi:10.1093/cvr/cvae190)
Supplement: cvae190_Supplementary_Data [file cvae190_supplementary_data.docx]

**SUPPLEMENTAL MATERIAL**

**Early Coronary Revascularization Among “Stable” Patients with Non-ST-Segment Elevation Acute Coronary Syndromes: The Role of Diabetes and Age**

Table of Contents

[**SUPPLEMENTAL METHODS** 3](#_Toc166750418)

[**Risk factors definitions** 3](#_Toc166750419)

[**Multiple Imputation using Chained Equation (MICE) algorithm** 3](#_Toc166750420)

[**Inverse Propensity Score Weighting Analysis** 3](#_Toc166750421)

[**Computation of Relative Risk and its Confidence Interval** 4](#_Toc166750422)

[**Comparison of means and prevalences in the weighted sample** 5](#_Toc166750423)

[**SUPPLEMENTAL RESULTS** 6](#_Toc166750424)

[**Figure S1**. Study flowchart. 6](#_Toc166750425)

[**Figure S2.** Boxplots representing crossover rates (panel A) and the timing of revascularization (panel B) in patients undergoing an initial conservative strategy 7](#_Toc166750426)

[**Figure S3.** Median difference in length of hospitalization stratified by early treatment strategy 8](#_Toc166750427)

[**Figure S4.** Distribution of stenotic lesions in patients with and without diabetes. 9](#_Toc166750428)

[**Figure S5.** Risk ratios of PCI complications (panel A and B) and major bleeding (panel C and D) stratified by diabetic status and treatment strategy. 10](#_Toc166750429)

[**Table S1.** Baseline characteristics of the overall population NSTE-ACS patients stratified by treatment strategy 11](#_Toc166750430)

[**Table S2.** Inverse probability weighting models: rates of missing values 13](#_Toc166750431)

[**Table S3.** General logistic regression and regression coefficients in the propensity score model in the overall NSTE-ACS population (early invasive vs initial conservative strategy) 14](#_Toc166750432)

[**Table S4.** Inverse probability of treatment weighting: clinical factors and outcomes stratified by treatment strategy in diabetic patients ≥65 years with NSTEMI 15](#_Toc166750433)

[**Table S5.** Inverse probability of treatment weighting: clinical factors and outcomes stratified by treatment strategy in diabetic patients ≥65 years with GRACE score >140 16](#_Toc166750434)

[**Table S6.** Inverse probability of treatment weighting: clinical factors and outcomes stratified by treatment strategy in nondiabetic patients ≥65 years with NSTEMI 17](#_Toc166750435)

[**Table S7.** Inverse probability of treatment weighting: clinical factors and outcomes stratified by treatment strategy in nondiabetic patients ≥65 years with GRACE score >140 18](#_Toc166750436)

[**Table S8.** Inverse probability of treatment weighting: clinical factors and outcomes stratified by treatment strategy in diabetic patients <65 years with NSTEMI 19](#_Toc166750437)

[**Table S9.** Inverse probability of treatment weighting: clinical factors and outcomes stratified by treatment strategy in diabetic patients <65 years with GRACE score >140 20](#_Toc166750438)

[**Table S10.** Inverse probability of treatment weighting: clinical factors and outcomes stratified by treatment strategy in nondiabetic patients <65 years with NSTEMI 21](#_Toc166750439)

[**Table S11.** Inverse probability of treatment weighting: clinical factors and outcomes stratified by treatment strategy in nondiabetic patients <65 years with GRACE score >140 22](#_Toc166750440)

[**Table S12.** Inverse probability of treatment weighting: clinical factors and outcomes stratified by diabetes in patients ≥65 years undergoing an early invasive strategy 23](#_Toc166750441)

[**Table S13.** Inverse probability of treatment weighting: clinical factors and outcomes stratified by diabetes in patients ≥65 years undergoing an initial conservative strategy 24](#_Toc166750442)

[**Table S14.** Inverse probability of treatment weighting: clinical factors and outcomes stratified by diabetes in patients <65 years undergoing an early invasive strategy 25](#_Toc166750443)

[**Table S15.** Inverse probability of treatment weighting: clinical factors and outcomes stratified by diabetes in patients <65 years undergoing an initial conservative strategy 26](#_Toc166750444)

[**Table S16.** Inverse probability of treatment weighting: complications in patients undergoing PCI stratified by diabetes status. 27](#_Toc166750445)

[**Table S17.** Inverse probability of treatment weighting: complications in diabetic patients undergoing PCI stratified by initial treatment strategy 28](#_Toc166750446)

[**Table S18.** Inverse probability of treatment weighting: complications in nondiabetic patients undergoing PCI stratified by initial treatment strategy 29](#_Toc166750447)

[**Table S19.** Inverse probability of treatment weighting: major bleeding stratified by diabetes status 30](#_Toc166750448)

[**Table S20.** Inverse probability of treatment weighting: major bleeding in diabetic patients stratified by initial treatment strategy 31](#_Toc166750449)

[**Table S21.** Inverse probability of treatment weighting: major bleeding in nondiabetic patients stratified by initial treatment strategy 32](#_Toc166750450)

[**Table S22.** Inverse probability of treatment weighting: clinical factors and outcomes stratified by age subgroup and treatment strategy in diabetic patients 33](#_Toc166750451)

[**Table S23.** Inverse probability of treatment weighting: clinical factors and outcomes stratified by age subgroup and treatment strategy in non-diabetic patients 35](#_Toc166750452)

[**REFERENCES** 38](#_Toc166750453)

# **SUPPLEMENTAL METHODS**

## **Risk factors definitions**

Smoking habits were self-reported. We defined current smokers as individuals who smoked 100 cigarettes in his or her lifetime and who smoked cigarettes, cigars, and cigarillos at the time of the index event. Everyday smokers or someday smokers were all included in this definition according to recommendations from the National Health Interview Survey^1^. Hypertension, hypercholesterolemia, and diabetes were assessed by designation of medical history prior to admission in the database. In addition, subjects taking medication because of arterial hypertension were classified to have arterial hypertension, even when blood pressure was controlled. Diabetes mellitus was also defined with regard to oral blood glucose lowering therapy or substitution of insulin. Positive family history of coronary artery disease (CAD) was defined as myocardial infarction, cardiac death, or need for coronary revascularization in a first-degree relative with early onset (under the age of 55 in men or 65 in women).

## **Multiple Imputation using Chained Equation (MICE) algorithm**

Multiple Imputation using Chained Equation (MICE) algorithm is an efficient and popular method to fill in missing data where each missing value on some records is replaced by a value obtained from related cases in the whole set of records. Thus, imputation for clinical features was conducted using the chained equations across other features^2^. More specifically, MICE algorithm sequentially imputes the missing values of clinical features based on both observed values and previously imputed values. This sequential imputation is conducted via chained equations.

We tried multiple imputations using the MICE algorithm for the initial analyses to address the uncertainty in the imputation process. More specifically, we generated multiple imputed datasets and check whether the conclusions are consistent across the different imputed datasets. If the conclusions are consistent across multiple imputed datasets, we use a single imputed dataset (by MICE algorithm) as the final dataset to report the results of statistical analyses in the paper.

## **Inverse Propensity Score Weighting Analysis**

We used Inverse Propensity Score Weighting (IPW) to balance the distribution of covariates between two patient groups. Note that we use Logistic Regression to estimate the propensity scores ({P}(Z=1 | x)). If *e* denotes the estimated propensity score (i.e. e=\hat{P}(Z=1 | x), where the patient x is included in patient group 1; then, 1-e = \hat{P}(Z=0 | x)), then the original sample is weighted by the following weights: Z/e+(1−Z)/ 1−e where Z represents the patient group. For instance, patients undergoing an early invasive strategy (Z=1) are assigned a weight equal to the reciprocal of the propensity score (1/e), while patients undergoing an initial conservative strategy (Z=0) are assigned a weight equal to the reciprocal of one minus the propensity score (1/1-e). The weighting procedure for each sample balances the covariate distributions between two patient groups^3^.

Inverse probability of treatment weighting method can potentially result in unstable and biased estimates if some of the weights are very high. To avoid excessive weights, we compared results with other methods for handling confounding. We included probability of treatment variables in a multivariable model. We also used XGBoost, a decision-tree-based ensemble machine learning algorithm, as an alternative multivariable model for estimating the probability of treatment. Conclusions from theses analyses were the same as our current results. Further, we created a threshold for weights to avoid the impacts of the outliers (we use 0.01 as threshold). Therefore, the inverse probability of treatment weighting analyses presented in the current analysis were quite stable.

## **Computation of Relative Risk and its Confidence Interval**

In a two-group cohort study, the risk ratio (RR, also called relative risk), is usually applied to compare risks of a health event between two independent binomial populations that differ by a demographic characteristic (i.e. sex, age) or by the level of exposure to a specific drug or risk factor. In such types of studies, data can be summarized in a confusion matrix as follows:

|  | **Risk of Designated Outcome** | |  |
| --- | --- | --- | --- |
|  | **Yes** | **No** | **Total** |
| **Exposed** | a | b | a+b (*H_1_*) |
| **Unexposed** | c | d | c+d (*H_0_*) |
| **Total** | a+c | b+d |  |

Where *H_1_* and *H_0_* correspond to the total number of exposed and unexposed patients, respectively, whereas *a* and *c* represent the number of exposed and unexposed patients at risk for the designated outcome, respectively.

RR is defined as the ratio between the risk of outcome in exposed patients (*H_1_*) and the risk of outcome in unexposed patients (*H_0,_*) which can be summarized as:

$$RR=\frac{\left( \frac{a}{H_{1}} \right)}{\left( \frac{c}{H_{0}} \right)}$$

When applying this equation to an IPTW balanced population, $\frac{a}{H_{1}}$ will be assigned a weight equal to the reciprocal of the propensity score ($\frac{1}{e}$) and $\frac{c}{H_{0}}$ will be weighted by the reciprocal of one minus the propensity score ($\frac{1}{(1-e)}$).

In order to compute the lower and upper (1-α) confidence limit RR_L_ for RR, we operate in the assumption of log normal distribution^4^. In particular, the variate $\log\frac{\left( \frac{a}{H_{1}} \right)}{\left( \frac{c}{H_{0}} \right)}$= $\log\frac{a}{H_{1}}- \log\frac{c}{H_{0}}$is approximately normally distributed with approximate mean log(RR) and estimated variance $\frac{1-\left( \frac{a}{H_{1}} \right)}{a}$ + $\frac{1-(\frac{c}{H_{0}})}{c}$ .

It follows that RR_L_ can be computed by solving the following equation:

$$\frac{\left[ log( \frac{\frac{a}{H_{1}}}{\frac{c}{H_{0}}})- \log({RR}_{L}) \right]}{\left[ \frac{1-\left( \frac{a}{H_{1}} \right)}{a} + \frac{1-(\frac{c}{H_{0}})}{c} \right]^{1/2}}=z_{1-\alpha}$$

Where $z_{1-\alpha}$, is the 100(1-α) percentage point of the N(O, 1) distribution

## **Comparison of means and prevalences in the weighted sample**

To evaluate the balance of the baseline covariate distributions between treatment and control groups, standardized difference (SD) is widely used in inverse probability of treatment weighting (IPTW) framework. For the baseline analysis, we use standard SD which is defined as follows: $\frac{m_{t}-m_{c}}{\sqrt{\frac{s_{t}^{2}+s_{c}^{2}}{2}}}$ for continuous variables and $\frac{m_{t}-m_{c}}{\sqrt{\frac{m_{t}(1-m_{t})+m_{c}(1-m_{c})}{2}}}$ for binary variable where $m_{t}, m_{c}$ are sample mean of the variables for treatment and control group, and $s_{t}^{2}, s_{c}^{2}$ are sample variance of the variables for treatment and control group, respectively. For IPTW analysis, we use weighted SD where $m_{t}, m_{c}$ are replaced to weighted sample mean of the variables for treatment and control group, and $s_{t}^{2}, s_{c}^{2}$ are replaced to weighted sample variance of the variables for treatment and control group, respectively. Weights are determined by the inverse probability of treatment received. In general, 0.1 is the reasonable threshold to determine whether two distributions are balanced (i.e., if SD > 0.1, the baseline covariate is imbalanced)^5^.

# **SUPPLEMENTAL RESULTS**

## **Figure S1**. Study flowchart.


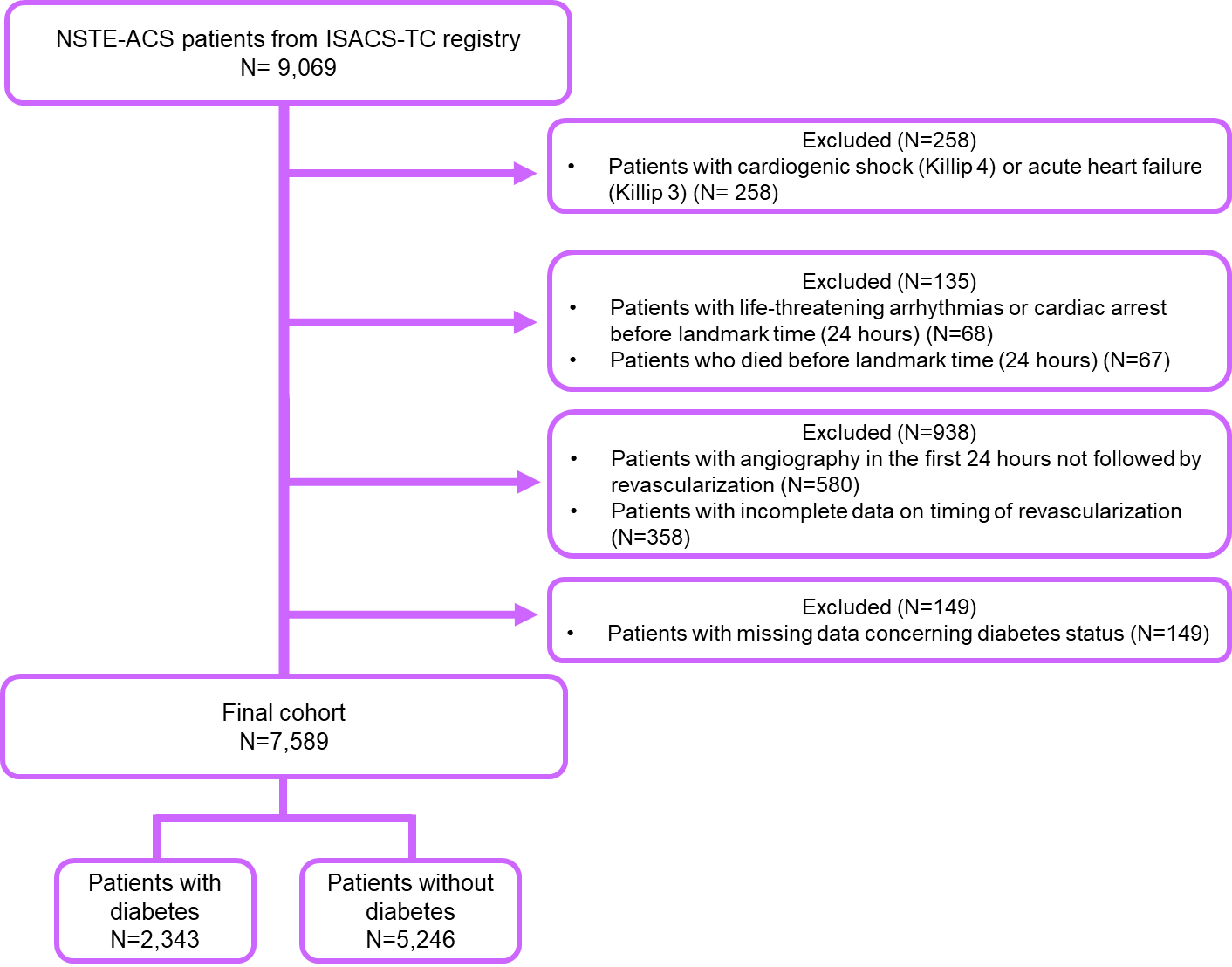


## **Figure S2.** Boxplots representing crossover rates (panel A) and the timing of revascularization (panel B) in patients undergoing an initial conservative strategy


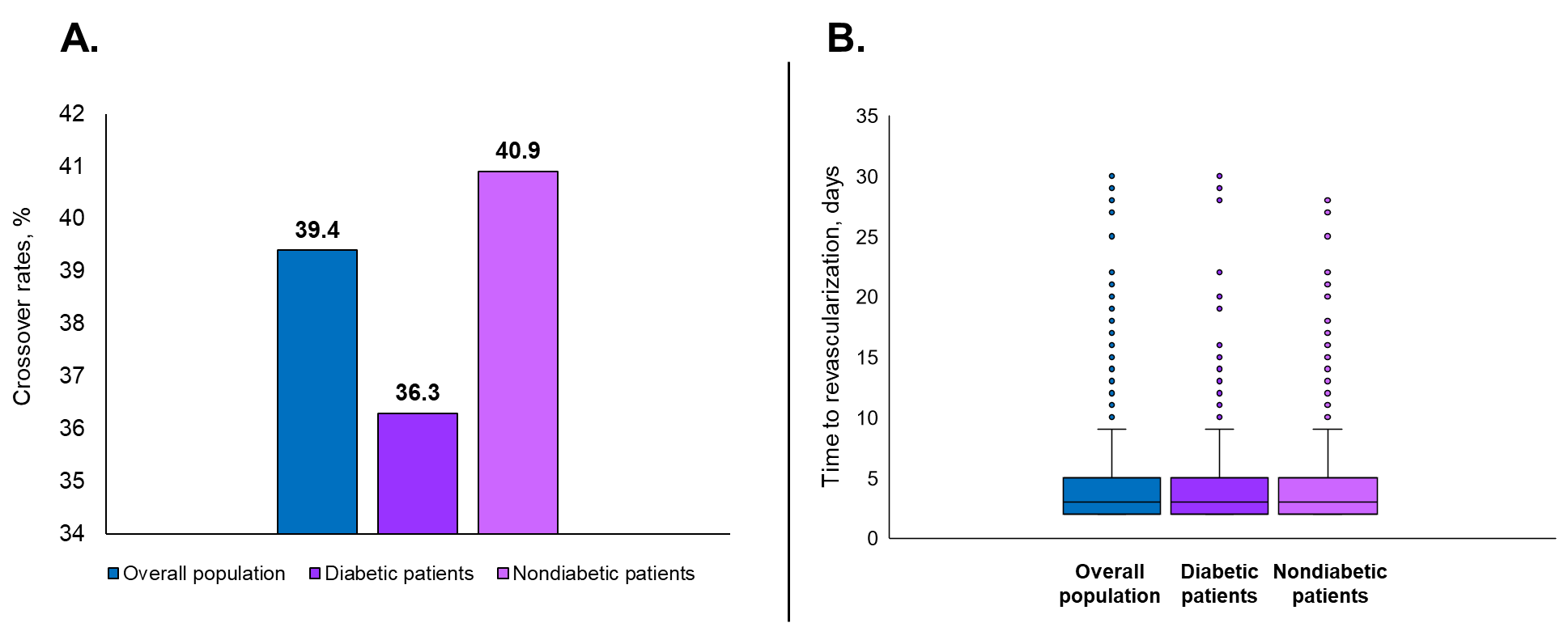


**Figure S3.** Median difference in length of hospitalization stratified by early treatment strategy.

**
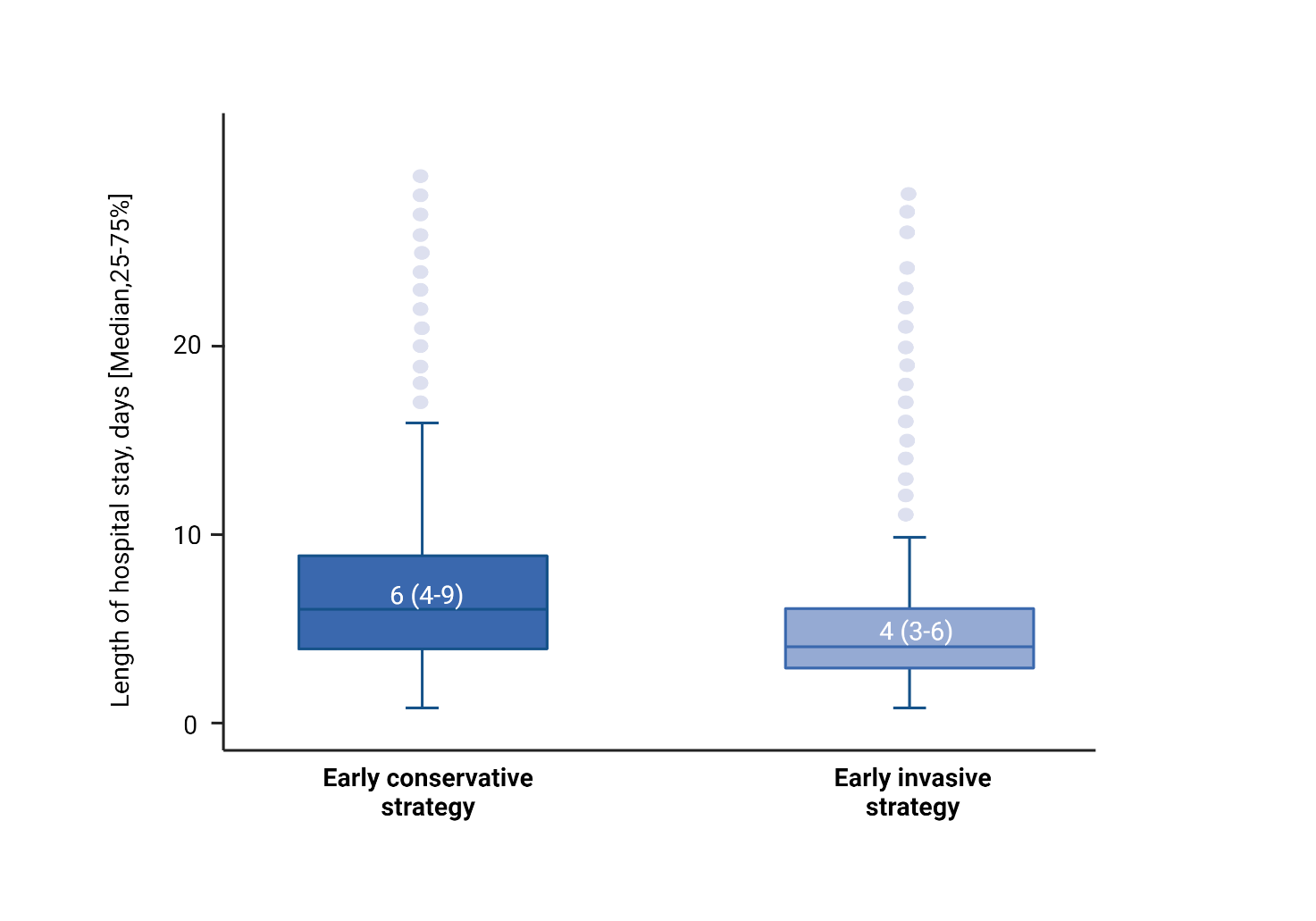
**

**Figure S4.** Distribution of stenotic lesions in patients with and without diabetes. Multivessel disease was defined as at least 2 main branches of the epicardial coronary artery with ≥70% stenotic lesions or ≥50% stenosis in the left main coronary artery.

**
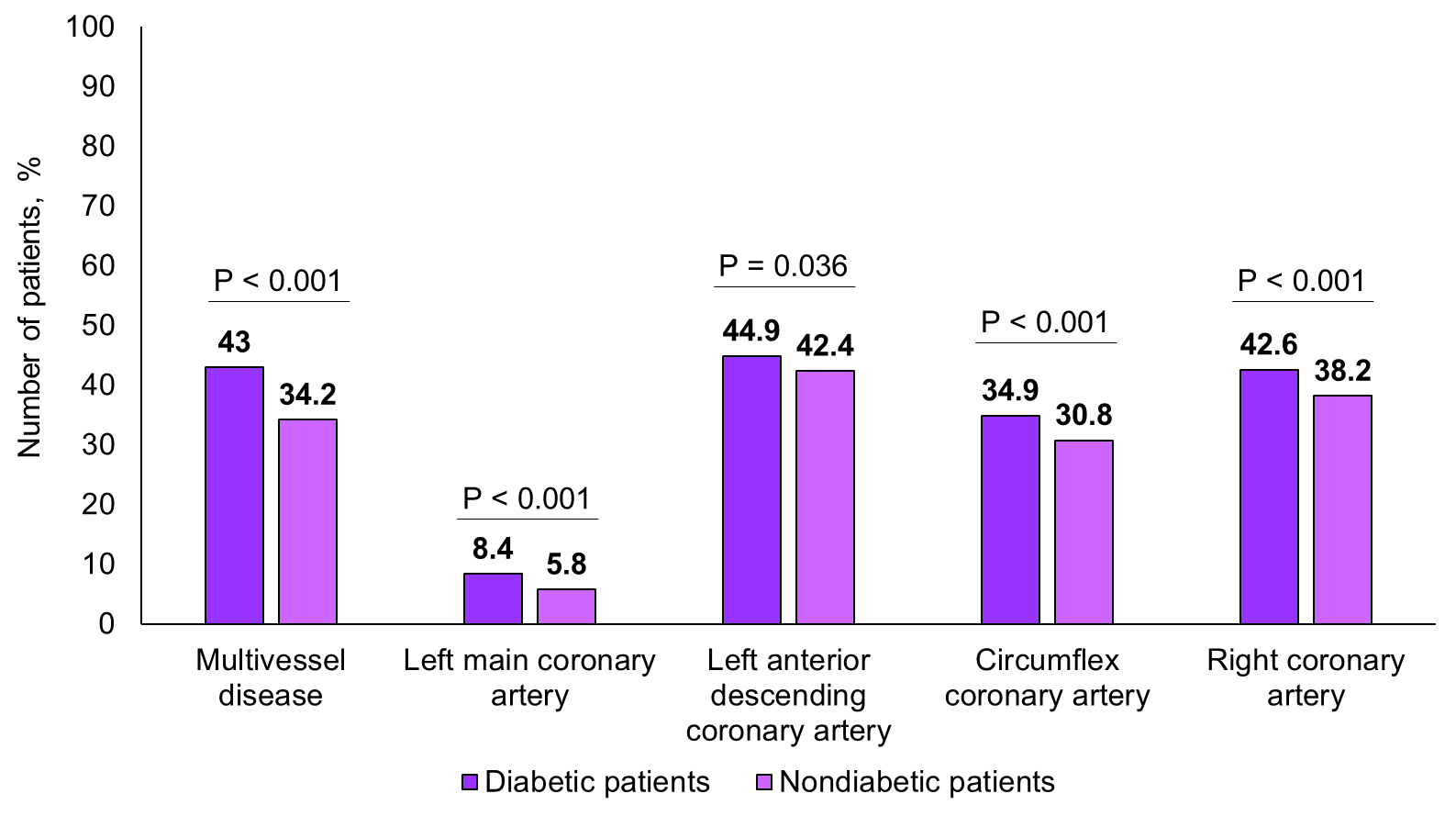
**

## **Figure S5.** Risk ratios of PCI complications (panel A and B) and major bleeding (panel C and D) stratified by diabetic status and treatment strategy.


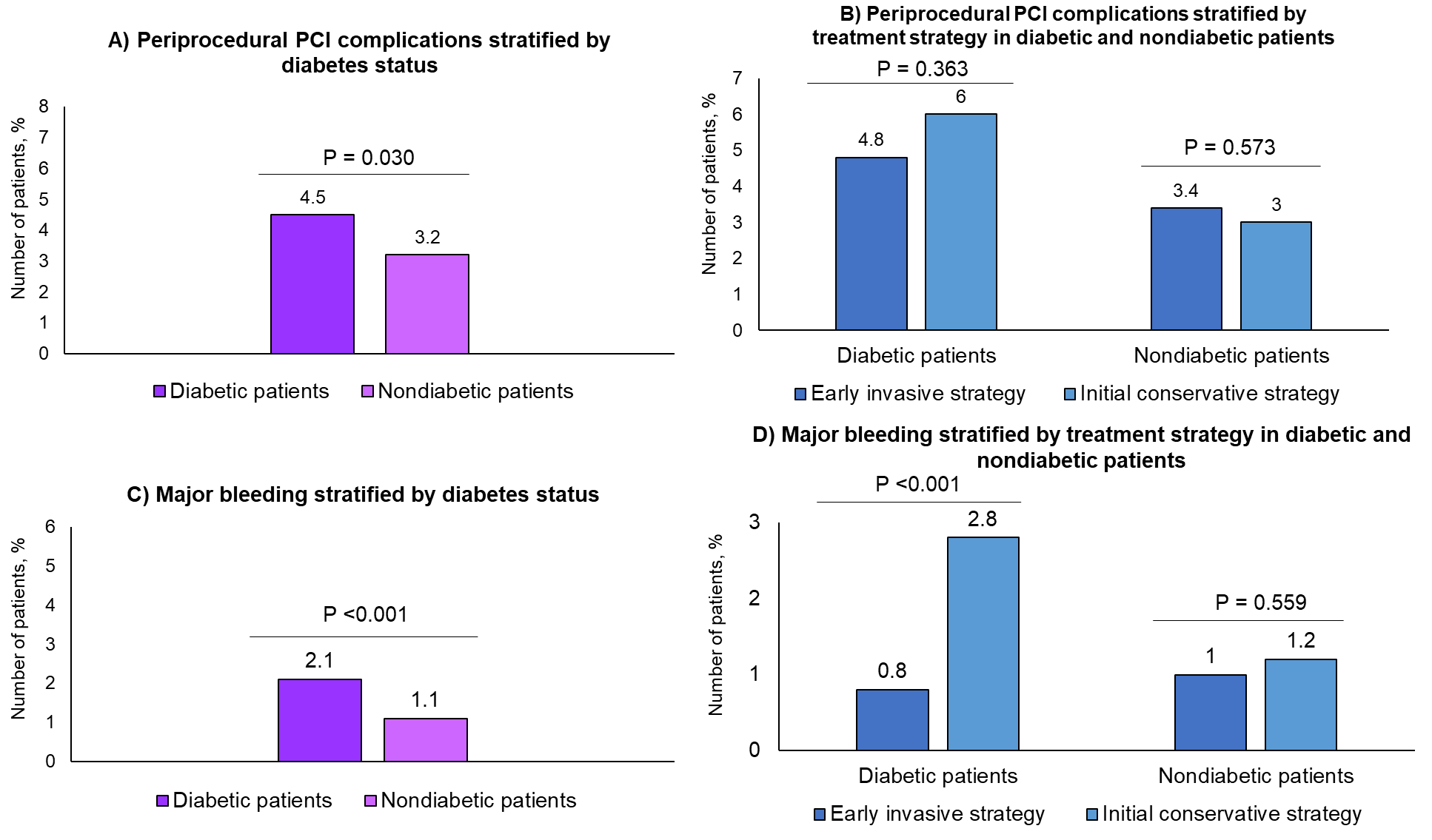


| **Table S1.** Baseline characteristics of the overall population NSTE-ACS patients stratified by treatment strategy | | | | | |
| --- | --- | --- | --- | --- | --- |
|  | **Overall population**  **N=7,589** | **Early invasive strategy**  **N=3,513** | **Initial conservative strategy**  **N=4,076** | **P value*** | **Standardized mean difference*** |
| Age | 64.4±11.6 | 63.2 ± 11.0 | 65.5 ± 12.1 | <0.001 | -0.20 |
| Women | 2,450 (32.3) | 995 (28.3) | 1,455 (35.7) | 0.001 | 0.07 |
| **Cardiovascular risk factors** | | | | | |
| Diabetes | 2,343 (30.9) | 1,016 (28.9) | 1,327 (32.6) | <0.001 | -0.07 |
| Hypercholesterolemia | 3,679 (48.5) | 1,773 (50.5) | 1,906 (46.8) | 0.001 | 0.07 |
| Hypertension | 5,900 (77.7) | 2,679 (76.3) | 3,221 (79.0) | 0.004 | -0.06 |
| Current smokers | 2,486 (32.8) | 1,307 (37.2) | 1,179 (28.9) | <0.001 | 0.17 |
| Family history of CAD | 2,647 (34.9) | 1,262 (35.9) | 1,385 (34.0) | 0.07 | 0.04 |
| **History of ischemic heart disease** | | | | | |
| Chronic coronary syndrome | 2,301 (30.3) | 934 (26.6) | 1,367 (33.5) | <0.001 | -0.15 |
| Prior myocardial infarction | 1,762 (23.2) | 765 (21.8) | 997 (24.5) | 0.05 | -0.06 |
| Prior CABG | 326 (4.3) | 97 (2.8) | 229 (5.6) | <0.001 | -0.14 |
| Prior PCI | 1,187 (15.6) | 635 (18.1) | 552 (13.5) | <0.001 | 0.12 |
| **History of cardiovascular disease** | | | | | |
| Peripheral artery disease | 281 (3.7) | 131 (3.7) | 150 (3.7) | 0.91 | 0.002 |
| Prior heart failure | 462 (6.1) | 159 (4.5) | 303 (7.4) | <0.001 | -0.12 |
| Prior stroke or TIA | 373 (4.9) | 131 (3.7) | 242 (5.9) | <0.001 | -0.10 |
| **Other comorbidities** |  |  |  |  |  |
| Chronic kidney disease | 674 (8.9) | 274 (7.8) | 400 (9.8) | 0.001 | -0.07 |
| **Clinical presentation on hospital admission** | | | | | |
| Heart rate, bpm | 82.2±19.9 | 79.8 ± 18.2 | 84.3 ± 21.0 | <0.001 | -0.22 |
| SBP, mmHg | 143.3±25.8 | 144.3 ± 25.6 | 142.5 ± 26.0 | 0.003 | 0.06 |
| NSTEMI | 5,927 (78.1) | 2,876 (81.9) | 3,051 (74.9) | <0.001 | 0.17 |
| UA | 1,647 (21.7) | 637 (18.1) | 1,010 (24.8) | <0.001 | -0.16 |
| **Medications taken before hospitalization** | | | | | |
| Antiplatelet medications | 3,548 (46.8) | 1,628 (46.3) | 1,920 (47.1) | 0.50 | -0.01 |
| ACE-inhibitors or ARBs | 4,075 (53.7) | 1,824 (51.9) | 2,251 (55.2) | 0.004 | -0.06 |
| Beta blockers | 3,408 (44.9) | 1,561 (44.4) | 1,847 (45.3) | 0.44 | -0.01 |
| Statins | 2,742 (36.1) | 1,312 (37.3) | 1,430 (35.1) | 0.04 | 0.04 |
| **Medications administered on hospital admission** | | | | | |
| Antiplatelet medications | 7,463 (98.3) | 3,494 (99.5) | 3,969 (97.4) | <0.001 | 0.16 |
| **Medications administered during hospitalization and at discharge** | | | | | |
| Beta blockers | 5,902 (77.8) | 2,661 (75.7) | 3,241 (79.5) | <0.001 | -0.09 |
| ACE inhibitors or ARBs | 6,025 (79.4) | 2,788 (79.4) | 3,237 (79.4) | 0.95 | -0.001 |
| Statins | 7,113 (93.7) | 3,381 (96.2) | 3,732 (91.6) | <0.001 | 0.19 |
| **Revascularization type** | | | | | |
| PCI | 4,689 (61.8) | 3,459 (98.5) | 1,230 (30.2) | <0.001 | 2.03 |
| CABG | 688 (9.1) | 281 (8.0) | 407 (10.0) | 0.002 | -0.06 |
| **In-hospital complications** | | | | | |
| Major bleeding | 112 (1.5) | 35 (1.0) | 77 (1.9) | 0.001 | -0.07 |
| PCI-related | 186 (2.5) | 130 (3.7) | 56 (1.4) | <0.001 | 0.14 |
| **Outcomes** |  |  |  | **P value** | |
| 30-day mortality | 225 (3.0) | 637 (18.1) | 1,148 (28.2) | <0.001 | |
| Risk ratio (95% CI) |  | 0.43 (0.32 – 0.58) | |  |  |
| *Calculated between early invasive strategy group and initial conservative strategy group  Data are expressed as mean ± standard deviation or n (%), unless otherwise specified.  Abbreviations: ACE= angiotensin converting enzyme; ARBs=angiotensin receptor blockers; bpm=beats per minute; CABG=coronary artery bypass graft; CAD= coronary artery disease; NSTE-ACS= Non-ST-segment elevation acute coronary syndromes; NSTEMI= Non-ST segment elevation myocardial infarction; PCI=percutaneous coronary intervention; SBP=systolic blood pressure; TIA= Transient ischemic attack; UA= unstable angina | | | | | |

| **Table S2.** Inverse probability weighting models: rates of missing values | |
| --- | --- |
| ***Variable name*** | ***Rate of missing values (%)*** |
| Age | 1.4% |
| Female sex | 0.2% |
| **Cardiovascular risk factors** |  |
| Hypercholesterolemia | 7.1% |
| Hypertension | 1.0% |
| Current smoking | 3.8% |
| Family history of CAD | 10.4% |
| **History of ischemic heart disease** |  |
| Chronic coronary syndrome | 0.0% |
| Prior myocardial infarction | 0.0% |
| Prior CABG | 0.0% |
| Prior PCI | 0.0% |
| **History of cardiovascular disease** |  |
| Peripheral artery disease | 0.0% |
| Prior heart failure | 0.0% |
| Prior stroke or TIA | 0.0% |
| **Other comorbidities** |  |
| Chronic kidney disease | 27.4% |
| **Clinical presentation on hospital admission** |  |
| Heart rate | 18.1% |
| Systolic blood pressure | 18.4% |
| Data are presented as percentages (%)  Abbreviations: CABG=coronary artery bypass graft; CAD= coronary artery disease; PCI=percutaneous coronary intervention; TIA= Transient ischemic attack | |

| **Table S3.** General logistic regression and regression coefficients in the propensity score model in the overall NSTE-ACS population (early invasive vs initial conservative strategy) | | | | |
| --- | --- | --- | --- | --- |
|  | **β** | **SE** | **T statistics** | ***p* value** |
| **Constant term (α)** | 1.0065 | 0.2356 | 4.2719 | <0.0001 |
| Age | -0.0753 | 0.0233 | -3.2391 | 0.001 |
| Female sex | -0.2516 | 0.0522 | -4.8225 | <0.001 |
| Diabetes | -0.0231 | 0.0531 | -0.4360 | 0.66 |
| Hypercholesterolemia | 0.1834 | 0.0498 | 3.6817 | 0.0002 |
| Hypertension | -0.0735 | 0.0605 | -1.2156 | 0.22 |
| Current smoking | 0.1973 | 0.0540 | 3.6523 | 0.0003 |
| Family history of CAD | 0.0665 | 0.0527 | 1.2605 | 0.20 |
| Chronic coronary syndrome | -0.3756 | 0.0562 | -6.6808 | <0.001 |
| Prior myocardial infarction | -0.2527 | 0.0623 | -4.0556 | 0.0001 |
| Prior CABG | -0.6803 | 0.297 | -5.2455 | <0.001 |
| Prior PCI | 0.5704 | 0.0739 | 7.7220 | <0.001 |
| Peripheral artery disease | 0.1096 | 0.1282 | 0.8550 | 0.39 |
| Prior heart failure | -0.2418 | 0.1056 | -2.2896 | 0.02 |
| Prior stroke or TIA | -0.4548 | 0.1152 | -3.9497 | 0.0001 |
| Heart rate | -0.0108 | 0.0013 | -8.5285 | <0.001 |
| Systolic blood pressure | 0.0023 | 0.0009 | 2.4091 | 0.01 |
| Chronic kidney disease | -0.0357 | 0.0879 | -0.4062 | 0.68 |
| Optimized regression coefficient (ß) and constant term(α) for the logistic regression  Abbreviations: CABG=coronary artery bypass graft; CAD= coronary artery disease; PCI=percutaneous coronary intervention; TIA= Transient ischemic attack | | | | |

| **Table S4.** Inverse probability of treatment weighting: clinical factors and outcomes stratified by treatment strategy in diabetic patients ≥65 years with NSTEMI | | | |
| --- | --- | --- | --- |
|  | **Early invasive strategy**  **N=488** | **Initial conservative strategy**  **N=665** | **Standardized mean difference** |
| Age, years | 73.5±5.5 | 73.6±6.5 | -0.01 |
| Women | 44.7 | 44.5 | 0.003 |
| **Cardiovascular risk factors** | | | |
| Hypercholesterolemia | 50.3 | 49.9 | 0.009 |
| Hypertension | 89.8 | 90.1 | -0.009 |
| Current smoking | 17.4 | 17.6 | -0.005 |
| Family history of CAD | 28.5 | 28.7 | -0.004 |
| **History of ischemic heart disease** | |  |  |
| Chronic coronary syndrome | 27.4 | 27.1 | 0.006 |
| Prior myocardial infarction | 27.1 | 27.9 | -0.01 |
| Prior CABG | 6.2 | 6.6 | -0.01 |
| Prior PCI | 16.7 | 16.7 | -0.001 |
| **History of cardiovascular disease** | |  |  |
| Peripheral artery disease | 5.4 | 5.8 | -0.01 |
| Prior heart failure | 10.2 | 10.0 | 0.007 |
| Prior stroke or TIA | 7.9 | 7.2 | 0.02 |
| **Other comorbidities** |  |  |  |
| Chronic kidney disease | 17.8 | 18.7 | -0.02 |
| **Clinical presentation on hospital admission** | |  |  |
| Heart rate, bpm | 84.9±20.7 | 85.1±20.7 | -0.005 |
| SBP, mmHg | 143.7±26.0 | 143.7±27.2 | -0.001 |
| **Outcomes** |  |  | **P value** |
| 30-day mortality | 3.5 | 7.7 | 0.003 |
| Risk ratio (95% CI) | 0.43 (0.25 – 0.76) | |  |
| Data are expressed as weighted mean ± standard deviation or weighted rate, unless otherwise specified.  Abbreviations: bpm=beats per minute; CABG=coronary artery bypass graft; CAD= coronary artery disease; PCI=percutaneous coronary intervention; SBP=systolic blood pressure; TIA= Transient ischemic attack. | | | |

| **Table S5.** Inverse probability of treatment weighting: clinical factors and outcomes stratified by treatment strategy in diabetic patients ≥65 years with GRACE score >140 | | | |
| --- | --- | --- | --- |
|  | **Early invasive strategy**  **N=257** | **Initial conservative strategy**  **N=480** | **Standardized mean difference** |
| Age, years | 76.1±5.4 | 76.2±6.4 | -0.01 |
| Women | 44.9 | 46.1 | -0.02 |
| **Cardiovascular risk factors** | | | |
| Hypercholesterolemia | 49.4 | 51.6 | -0.04 |
| Hypertension | 88.0 | 90.2 | -0.06 |
| Current smoking | 14.7 | 15.0 | -0.008 |
| Family history of CAD | 30.1 | 31.7 | -0.03 |
| **History of ischemic heart disease** | |  |  |
| Chronic coronary syndrome | 41.6 | 38.4 | 0.06 |
| Prior myocardial infarction | 25.7 | 28.7 | -0.06 |
| Prior CABG | 7.0 | 7.5 | -0.02 |
| Prior PCI | 18.5 | 19.2 | -0.01 |
| **History of cardiovascular disease** | |  |  |
| Peripheral artery disease | 5.7 | 7.0 | -0.05 |
| Prior heart failure | 14.8 | 14.1 | 0.02 |
| Prior stroke or TIA | 9.5 | 8.0 | 0.05 |
| **Other comorbidities** |  |  |  |
| Chronic kidney disease | 21.5 | 24.7 | -0.07 |
| **Clinical presentation on hospital admission** | |  |  |
| Heart rate, bpm | 93.8±28.8 | 92.8±22.5 | 0.01 |
| SBP, mmHg | 133.8±28.1 | 134.9±27.8 | -0.03 |
| **Outcomes** |  |  | **P value** |
| 30-day mortality | 5.0 | 10.3 | 0.016 |
| Risk ratio (95% CI) | 0.46 (0.25 – 0.87) | |  |
| Data are expressed as weighted mean ± standard deviation or weighted rate, unless otherwise specified.  Abbreviations: bpm=beats per minute; CABG=coronary artery bypass graft; CAD= coronary artery disease; PCI=percutaneous coronary intervention; SBP=systolic blood pressure; TIA= Transient ischemic attack. | | | |

| **Table S6.** Inverse probability of treatment weighting: clinical factors and outcomes stratified by treatment strategy in nondiabetic patients ≥65 years with NSTEMI | | | |
| --- | --- | --- | --- |
|  | **Early invasive strategy**  **N=859** | **Initial conservative strategy**  **N=993** | **Standardized mean difference** |
| Age, years | 74.2±6.3 | 74.2±6.5 | -0.01 |
| Women | 36.0 | 35.7 | 0.005 |
| **Cardiovascular risk factors** | | | |
| Hypercholesterolemia | 42.6 | 41.8 | 0.01 |
| Hypertension | 82.4 | 82.3 | 0.001 |
| Current smoking | 21.9 | 21.9 | -0.0006 |
| Family history of CAD | 28.3 | 28.0 | 0.006 |
| **History of ischemic heart disease** | |  |  |
| Chronic coronary syndrome | 25.1 | 25.5 | -0.009 |
| Prior myocardial infarction | 22.2 | 21.1 | 0.02 |
| Prior CABG | 5.2 | 4.7 | 0.02 |
| Prior PCI | 12.1 | 12.0 | 0.003 |
| **History of cardiovascular disease** | |  |  |
| Peripheral artery disease | 4.6 | 4.4 | 0.006 |
| Prior heart failure | 6.4 | 6.7 | -0.01 |
| Prior stroke or TIA | 7.0 | 6.5 | 0.01 |
| **Other comorbidities** |  |  |  |
| Chronic kidney disease | 10.7 | 10.4 | 0.008 |
| **Clinical presentation on hospital admission** | |  |  |
| Heart rate, bpm | 82.5±20.9 | 82.8±21.9 | -0.009 |
| SBP, mmHg | 142.2±25.7 | 142.4±25.3 | -0.008 |
| **Outcomes** |  |  | **P value** |
| 30-day mortality | 2.8 | 5.7 | 0.003 |
| Risk ratio (95% CI) | 0.48 (0.30 – 0.78) | |  |
| Data are expressed as weighted mean ± standard deviation or weighted rate, unless otherwise specified.  Abbreviations: bpm=beats per minute; CABG=coronary artery bypass graft; CAD= coronary artery disease; PCI=percutaneous coronary intervention; SBP=systolic blood pressure; TIA= Transient ischemic attack. | | | |

| **Table S7.** Inverse probability of treatment weighting: clinical factors and outcomes stratified by treatment strategy in nondiabetic patients ≥65 years with GRACE score >140 | | | |
| --- | --- | --- | --- |
|  | **Early invasive strategy**  **N=464** | **Initial conservative strategy**  **N=685** | **Standardized mean difference** |
| Age, years | 76.8±6.7 | 76.9±6.4 | -0.01 |
| Women | 38.5 | 37.8 | 0.01 |
| **Cardiovascular risk factors** | | | |
| Hypercholesterolemia | 47.1 | 45.3 | 0.03 |
| Hypertension | 82.6 | 82.7 | -0.002 |
| Current smoking | 16.1 | 16.2 | -0.002 |
| Family history of CAD | 33.2 | 32.3 | 0.01 |
| **History of ischemic heart disease** | |  |  |
| Chronic coronary syndrome | 37.3 | 37.4 | -0.002 |
| Prior myocardial infarction | 24.8 | 23.5 | 0.03 |
| Prior CABG | 6.3 | 5.9 | 0.01 |
| Prior PCI | 15.2 | 14.8 | 0.01 |
| **History of cardiovascular disease** | |  |  |
| Peripheral artery disease | 6.1 | 6.1 | -0.0004 |
| Prior heart failure | 10.0 | 10.6 | -0.01 |
| Prior stroke or TIA | 8.9 | 8.2 | 0.02 |
| **Other comorbidities** |  |  |  |
| Chronic kidney disease | 16.1 | 15.4 | 0.01 |
| **Clinical presentation on hospital admission** | |  |  |
| Heart rate, bpm | 87.9±26.5 | 88.0±23.9 | -0.003 |
| SBP, mmHg | 133.2±26.6 | 133.9±26.1 | -0.02 |
| **Outcomes** |  |  | **P value** |
| 30-day mortality | 3.5 | 7.6 | 0.005 |
| Risk ratio (95% CI) | 0.45 (0.25 – 0.79) | |  |
| Data are expressed as weighted mean ± standard deviation or weighted rate, unless otherwise specified.  Abbreviations: bpm=beats per minute; CABG=coronary artery bypass graft; CAD= coronary artery disease; PCI=percutaneous coronary intervention; SBP=systolic blood pressure; TIA= Transient ischemic attack. | | | |

| **Table S8.** Inverse probability of treatment weighting: clinical factors and outcomes stratified by treatment strategy in diabetic patients <65 years with NSTEMI | | | |
| --- | --- | --- | --- |
|  | **Early invasive strategy**  **N=345** | **Initial conservative strategy**  **N=351** | **Standardized mean difference** |
| Age, years | 56.6±6.0 | 56.5±7.0 | 0.01 |
| Women | 29.7 | 30.0 | -0.006 |
| **Cardiovascular risk factors** | | | |
| Hypercholesterolemia | 51.0 | 50.8 | 0.002 |
| Hypertension | 80.2 | 80.1 | 0.002 |
| Current smoking | 40.3 | 40.6 | -0.006 |
| Family history of CAD | 37.8 | 37.0 | 0.01 |
| **History of ischemic heart disease** | |  |  |
| Chronic coronary syndrome | 25.0 | 24.4 | 0.01 |
| Prior myocardial infarction | 23.4 | 22.6 | 0.01 |
| Prior CABG | 5.8 | 5.1 | 0.03 |
| Prior PCI | 15.6 | 14.7 | 0.02 |
| **History of cardiovascular disease** | |  |  |
| Peripheral artery disease | 3.0 | 3.3 | -0.01 |
| Prior heart failure | 6.0 | 6.1 | -0.001 |
| Prior stroke or TIA | 4.7 | 4.6 | 0.004 |
| **Other comorbidities** |  |  |  |
| Chronic kidney disease | 8.1 | 7.2 | 0.03 |
| **Clinical presentation on hospital admission** | |  |  |
| Heart rate, bpm | 86.3±23.4 | 85.4±19.7 | 0.03 |
| SBP, mmHg | 146.2±27.0 | 146.3±25.6 | -0.004 |
| **Outcomes** |  |  | **P value** |
| 30-day mortality | 1.4 | 2.0 | 0.519 |
| Risk ratio (95% CI) | 0.68 (0.21 – 2.21) | |  |
| Data are expressed as weighted mean ± standard deviation or weighted rate, unless otherwise specified.  Abbreviations: bpm=beats per minute; CABG=coronary artery bypass graft; CAD= coronary artery disease; PCI=percutaneous coronary intervention; SBP=systolic blood pressure; TIA= Transient ischemic attack. | | | |

| **Table S9.** Inverse probability of treatment weighting: clinical factors and outcomes stratified by treatment strategy in diabetic patients <65 years with GRACE score >140 | | | |
| --- | --- | --- | --- |
|  | **Early invasive strategy**  **N=36** | **Initial conservative strategy**  **N=78** | **Standardized mean difference** |
| Age, years | 60.2±3.7 | 60.1±3.5 | 0.03 |
| Women | 18.8 | 20.8 | -0.06 |
| **Cardiovascular risk factors** | | | |
| Hypercholesterolemia | 53.0 | 52.7 | 0.005 |
| Hypertension | 86.4 | 52.5 | 0.06 |
| Current smoking | 20.5 | 22.8 | -0.05 |
| Family history of CAD | 50.3 | 52.1 | -0.03 |
| **History of ischemic heart disease** | |  |  |
| Chronic coronary syndrome | 55.6 | 51.4 | 0.08 |
| Prior myocardial infarction | 33.3 | 34.9 | -0.03 |
| Prior CABG | 18.4 | 18.3 | 0.001 |
| Prior PCI | 28.9 | 24.7 | 0.08 |
| **History of cardiovascular disease** | |  |  |
| Peripheral artery disease | 13.4 | 10.7 | 0.08 |
| Prior heart failure | 10.9 | 11.9 | -0.03 |
| Prior stroke or TIA | 11.8 | 8.6 | 0.09 |
| **Other comorbidities** |  |  |  |
| Chronic kidney disease | 23.9 | 22.5 | 0.03 |
| **Clinical presentation on hospital admission** | |  |  |
| Heart rate, bpm | 97.5±31.9 | 99.8±29.3 | -0.07 |
| SBP, mmHg | 126.4±33.9 | 127.4±29.3 | -0.03 |
| **Outcomes** |  |  | **P value** |
| 30-day mortality | 2.2 | 3.9 | 0.647 |
| Risk ratio (95% CI) | 0.56 (0.05 – 6.78) | |  |
| Data are expressed as weighted mean ± standard deviation or weighted rate, unless otherwise specified.  Abbreviations: bpm=beats per minute; CABG=coronary artery bypass graft; CAD= coronary artery disease; PCI=percutaneous coronary intervention; SBP=systolic blood pressure; TIA= Transient ischemic attack. | | | |

| **Table S10.** Inverse probability of treatment weighting: clinical factors and outcomes stratified by treatment strategy in nondiabetic patients <65 years with NSTEMI | | | |
| --- | --- | --- | --- |
|  | **Early invasive strategy**  **N=1,185** | **Initial conservative strategy**  **N=1,042** | **Standardized mean difference** |
| Age, years | 54.3±7.3 | 54.3±7.3 | 0.003 |
| Women | 23.4 | 23.4 | 0.0008 |
| **Cardiovascular risk factors** | | | |
| Hypercholesterolemia | 43.2 | 43.0 | 0.004 |
| Hypertension | 66.1 | 65.8 | 0.007 |
| Current smoking | 52.9 | 52.8 | 0.001 |
| Family history of CAD | 34.3 | 34.4 | -0.003 |
| **History of ischemic heart disease** | |  |  |
| Chronic coronary syndrome | 16.6 | 16.5 | 0.001 |
| Prior myocardial infarction | 18.8 | 18.7 | 0.0009 |
| Prior CABG | 1.4 | 1.4 | 0.005 |
| Prior PCI | 12.8 | 12.6 | 0.005 |
| **History of cardiovascular disease** | |  |  |
| Peripheral artery disease | 1.7 | 1.5 | 0.01 |
| Prior heart failure | 2.3 | 2.3 | 0.0007 |
| Prior stroke or TIA | 3.4 | 3.2 | 0.01 |
| **Other comorbidities** |  |  |  |
| Chronic kidney disease | 3.2 | 3.1 | 0.003 |
| **Clinical presentation on hospital admission** | |  |  |
| Heart rate, bpm | 81.2±18.4 | 81.3±18.6 | -0.003 |
| SBP, mmHg | 145.1±25.9 | 144.9±24.5 | 0.006 |
| **Outcomes** |  |  | **P value** |
| 30-day mortality | 1.2 | 1.7 | 0.35 |
| Risk ratio (95% CI) | 0.72 (0.35 – 1.45) | |  |
| Data are expressed as weighted mean ± standard deviation or weighted rate, unless otherwise specified.  Abbreviations: bpm=beats per minute; CABG=coronary artery bypass graft; CAD= coronary artery disease; PCI=percutaneous coronary intervention; SBP=systolic blood pressure; TIA= Transient ischemic attack. | | | |

| **Table S11.** Inverse probability of treatment weighting: clinical factors and outcomes stratified by treatment strategy in nondiabetic patients <65 years with GRACE score >140 | | | |
| --- | --- | --- | --- |
|  | **Early invasive strategy**  **N=105** | **Initial conservative strategy**  **N=93** | **Standardized mean difference** |
| Age, years | 59.2±4.3 | 59.1±4.7 | 0.02 |
| Women | 24.8 | 25.5 | -0.01 |
| **Cardiovascular risk factors** | | | |
| Hypercholesterolemia | 54.7 | 53.1 | 0.03 |
| Hypertension | 67.9 | 69.8 | -0.04 |
| Current smoking | 45.7 | 47.2 | -0.03 |
| Family history of CAD | 50.2 | 48.3 | 0.03 |
| **History of ischemic heart disease** | |  |  |
| Chronic coronary syndrome | 40.9 | 40.0 | 0.01 |
| Prior myocardial infarction | 19.4 | 19.4 | 0.001 |
| Prior CABG | 4.2 | 2.7 | 0.08 |
| Prior PCI | 23.0 | 22.4 | 0.01 |
| **History of cardiovascular disease** | |  |  |
| Peripheral artery disease | 2.5 | 2.3 | 0.01 |
| Prior heart failure | 8.9 | 7.6 | 0.04 |
| Prior stroke or TIA | 0.9 | 1.0 | -0.001 |
| **Other comorbidities** |  |  |  |
| Chronic kidney disease | 10.1 | 9.9 | 0.006 |
| **Clinical presentation on hospital admission** | |  |  |
| Heart rate, bpm | 97.0±29.8 | 96.2±26.6 | 0.02 |
| SBP, mmHg | 121.6±23.5 | 120.2±22.9 | 0.05 |
| **Outcomes** |  |  | **P value** |
| 30-day mortality | 5.1 | 2.5 | 0.35 |
| Risk ratio (95% CI) | 2.09 (0.44 – 9.99) | |  |
| Data are expressed as weighted mean ± standard deviation or weighted rate, unless otherwise specified.  Abbreviations: bpm=beats per minute; CABG=coronary artery bypass graft; CAD= coronary artery disease; PCI=percutaneous coronary intervention; SBP=systolic blood pressure; TIA= Transient ischemic attack. | | | |

| **Table S12.** Inverse probability of treatment weighting: clinical factors and outcomes stratified by diabetes in patients ≥65 years undergoing an early invasive strategy | | | |
| --- | --- | --- | --- |
|  | **Diabetic patients**  **N=588** | **Nondiabetic patients**  **N=1,048** | **Standardized mean difference** |
| Age, years | 72.8±5.3 | 72.8±5.6 | 0.005 |
| Women | 35.1 | 35.0 | 0.004 |
| **Cardiovascular risk factors** | | | |
| Hypercholesterolemia | 49.8 | 50.8 | -0.01 |
| Hypertension | 84.5 | 84.5 | 0.0000 |
| Current smoking | 21.3 | 21.0 | 0.007 |
| Family history of CAD | 31.8 | 33.0 | -0.02 |
| **History of ischemic heart disease** | |  |  |
| Chronic coronary syndrome | 30.1 | 30.0 | 0.0006 |
| Prior myocardial infarction | 24.2 | 24.9 | -0.01 |
| Prior CABG | 3.7 | 3.6 | 0.0005 |
| Prior PCI | 18.8 | 19.5 | -0.01 |
| **History of cardiovascular disease** | |  |  |
| Peripheral artery disease | 4.9 | 5.1 | -0.008 |
| Prior heart failure | 5.9 | 6.0 | -0.002 |
| Prior stroke or TIA | 5.8 | 5.7 | 0.002 |
| **Other comorbidities** |  |  |  |
| Chronic kidney disease | 11.8 | 11.9 | -0.001 |
| **Clinical presentation on hospital admission** | |  |  |
| Heart rate, bpm | 80.4±18.4 | 80.1±19.1 | 0.01 |
| SBP, mmHg | 144.4±24.5 | 144.2±25.7 | 0.007 |
| **Outcomes** |  |  | **P value** |
| 30-day mortality | 3.3 | 2.6 | 0.42 |
| Risk ratio (95% CI) | 1.27 (0.70 – 2.30) | |  |
| Data are expressed as weighted mean ± standard deviation or weighted rate, unless otherwise specified.  Abbreviations: bpm=beats per minute; CABG=coronary artery bypass graft; CAD= coronary artery disease; PCI=percutaneous coronary intervention; SBP=systolic blood pressure; TIA= Transient ischemic attack. | | | |

| **Table S13.** Inverse probability of treatment weighting: clinical factors and outcomes stratified by diabetes in patients ≥65 years undergoing an initial conservative strategy | | | |
| --- | --- | --- | --- |
|  | **Diabetic patients**  **N=856** | **Nondiabetic patients**  **N=1,306** | **Standardized mean difference** |
| Age, years | 74.9±6.7 | 74.9±6.6 | 0.002 |
| Women | 42.1 | 42.3 | -0.003 |
| **Cardiovascular risk factors** | | | |
| Hypercholesterolemia | 46.5 | 46.4 | 0.002 |
| Hypertension | 86.3 | 86.3 | -0.0001 |
| Current smoking | 16.0 | 15.8 | 0.004 |
| Family history of CAD | 28.4 | 28.4 | 0.0005 |
| **History of ischemic heart disease** | |  |  |
| Chronic coronary syndrome | 37.5 | 37.2 | 0.005 |
| Prior myocardial infarction | 25.7 | 25.8 | -0.001 |
| Prior CABG | 7.4 | 7.3 | 0.003 |
| Prior PCI | 13.3 | 13.2 | 0.002 |
| **History of cardiovascular disease** | |  |  |
| Peripheral artery disease | 5.4 | 5.4 | 0.001 |
| Prior heart failure | 10.3 | 10.4 | -0.0007 |
| Prior stroke or TIA | 7.8 | 8.0 | -0.008 |
| **Other comorbidities** |  |  |  |
| Chronic kidney disease | 14.9 | 14.9 | -0.0006 |
| **Clinical presentation on hospital admission** | |  |  |
| Heart rate, bpm | 85.5±20.9 | 85.7±24.2 | -0.007 |
| SBP, mmHg | 141.5±26.8 | 141.3±26.1 | 0.005 |
| **Outcomes** |  |  | **P value** |
| 30-day mortality | 7.3 | 5.2 | 0.05 |
| Risk ratio (95% CI) | 1.42 (1.00 – 2.03) | |  |
| Data are expressed as weighted mean ± standard deviation or weighted rate, unless otherwise specified.  Abbreviations: bpm=beats per minute; CABG=coronary artery bypass graft; CAD= coronary artery disease; PCI=percutaneous coronary intervention; SBP=systolic blood pressure; TIA= Transient ischemic attack. | | | |

| **Table S14.** Inverse probability of treatment weighting: clinical factors and outcomes stratified by diabetes in patients <65 years undergoing an early invasive strategy | | | |
| --- | --- | --- | --- |
|  | **Diabetic patients**  **N=428** | **Nondiabetic patients**  **N=1,449** | **Standardized mean difference** |
| Age, years | 55.0±6.4 | 54.9±7.0 | 0.02 |
| Women | 22.6 | 22.5 | 0.002 |
| **Cardiovascular risk factors** | | | |
| Hypercholesterolemia | 49.7 | 50.0 | -0.006 |
| Hypertension | 69.0 | 69.0 | -0.0007 |
| Current smoking | 51.3 | 51.3 | 0.001 |
| Family history of CAD | 37.7 | 38.1 | -0.008 |
| **History of ischemic heart disease** | |  |  |
| Chronic coronary syndrome | 23.8 | 23.9 | -0.002 |
| Prior myocardial infarction | 18.5 | 18.9 | -0.009 |
| Prior CABG | 1.9 | 1.9 | 0.004 |
| Prior PCI | 16.9 | 16.5 | 0.01 |
| **History of cardiovascular disease** | |  |  |
| Peripheral artery disease | 2.4 | 2.4 | 0.0004 |
| Prior heart failure | 3.5 | 3.4 | 0.005 |
| Prior stroke or TIA | 2.3 | 2.3 | 0.0002 |
| **Other comorbidities** |  |  |  |
| Chronic kidney disease | 3.7 | 3.8 | -0.003 |
| **Clinical presentation on hospital admission** | |  |  |
| Heart rate, bpm | 79.9±17.0 | 79.7±18.1 | 0.01 |
| SBP, mmHg | 143.4±25.1 | 144.2±25.9 | -0.03 |
| **Outcomes** |  |  | **P value** |
| 30-day mortality | 1.0 | 1.0 | 0.99 |
| Risk ratio (95% CI) | 0.99 (0.34 – 2.94) | |  |
| Data are expressed as weighted mean ± standard deviation or weighted rate, unless otherwise specified.  Abbreviations: bpm=beats per minute; CABG=coronary artery bypass graft; CAD= coronary artery disease; PCI=percutaneous coronary intervention; SBP=systolic blood pressure; TIA= Transient ischemic attack. | | | |

| **Table S15.** Inverse probability of treatment weighting: clinical factors and outcomes stratified by diabetes in patients <65 years undergoing an initial conservative strategy | | | |
| --- | --- | --- | --- |
|  | **Diabetic patients**  **N=471** | **Nondiabetic patients**  **N=1,443** | **Standardized mean difference** |
| Age, years | 54.4±8.4 | 55.0±7.2 | -0.07 |
| Women | 27.1 | 28.2 | -0.02 |
| **Cardiovascular risk factors** | | | |
| Hypercholesterolemia | 47.2 | 47.2 | 0.0003 |
| Hypertension | 71.1 | 71.0 | 0.002 |
| Current smoking | 46.2 | 44.1 | 0.04 |
| Family history of CAD | 38.9 | 40.5 | -0.03 |
| **History of ischemic heart disease** | |  |  |
| Chronic coronary syndrome | 29.2 | 29.8 | -0.01 |
| Prior myocardial infarction | 22.1 | 23.0 | -0.02 |
| Prior CABG | 3.8 | 3.9 | -0.003 |
| Prior PCI | 13.3 | 13.7 | -0.01 |
| **History of cardiovascular disease** | |  |  |
| Peripheral artery disease | 1.9 | 2.0 | -0.009 |
| Prior heart failure | 4.0 | 4.1 | -0.008 |
| Prior stroke or TIA | 3.4 | 3.8 | -0.02 |
| **Other comorbidities** |  |  |  |
| Chronic kidney disease | 4.0 | 4.0 | -0.001 |
| **Clinical presentation on hospital admission** | |  |  |
| Heart rate, bpm | 82.8±17.4 | 82.9±20.3 | -0.005 |
| SBP, mmHg | 143.4±25.9 | 143.7±25.3 | -0.01 |
| **Outcomes** |  |  | **P value** |
| 30-day mortality | 1.3 | 1.5 | 0.69 |
| Risk ratio (95% CI) | 0.84 (0.34 – 2.07) | |  |
| Data are expressed as weighted mean ± standard deviation or weighted rate, unless otherwise specified.  Abbreviations: bpm=beats per minute; CABG=coronary artery bypass graft; CAD= coronary artery disease; PCI=percutaneous coronary intervention; SBP=systolic blood pressure; TIA= Transient ischemic attack. | | | |

| **Table S16.** Inverse probability of treatment weighting: complications in patients undergoing PCI stratified by diabetes status. | | | |
| --- | --- | --- | --- |
|  | **Diabetic patients**  **N=1,361** | **Nondiabetic patients**  **N=3,338** | **Standardized mean difference** |
| Age, years | 63.3±10.6 | 63.2±11.4 | 0.008 |
| Women | 28.0 | 28.1 | -0.001 |
| **Cardiovascular risk factors** | | | |
| Hypercholesterolemia | 49.1 | 49.4 | -0.006 |
| Hypertension | 76.4 | 76.6 | -0.003 |
| Current smoking | 37.1 | 37.2 | -0.002 |
| Family history of CAD | 33.9 | 34.4 | -0.01 |
| **History of ischemic heart disease** | |  |  |
| Chronic coronary syndrome | 27.3 | 27.7 | -0.008 |
| Prior myocardial infarction | 21.3 | 21.7 | -0.008 |
| Prior CABG | 3.6 | 3.6 | 0.0002 |
| Prior PCI | 16.8 | 16.8 | 0.0008 |
| **History of cardiovascular disease** | |  |  |
| Peripheral artery disease | 3.8 | 3.7 | 0.008 |
| Prior heart failure | 5.0 | 5.1 | -0.0003 |
| Prior stroke or TIA | 4.0 | 4.3 | -0.01 |
| **Other comorbidities** |  |  |  |
| Chronic kidney disease | 7.7 | 7.9 | -0.006 |
| **Clinical presentation on hospital admission** | |  |  |
| Heart rate, bpm | 80.5±17.4 | 80.1±19.2 | 0.02 |
| SBP, mmHg | 144.3±243 | 144.7±25.5 | -0.01 |
| **Outcomes** |  |  | **P value** |
| PCI complications | 4.5 | 3.2 | 0.03 |
| Risk ratio (95% CI) | 1.43 (1.03 – 1.96) | |  |
| Data are expressed as weighted mean ± standard deviation or weighted rate, unless otherwise specified.  Abbreviations: bpm=beats per minute; CABG=coronary artery bypass graft; CAD= coronary artery disease; PCI=percutaneous coronary intervention; SBP=systolic blood pressure; TIA= Transient ischemic attack. | | | |

| **Table S17.** Inverse probability of treatment weighting: complications in diabetic patients undergoing PCI stratified by initial treatment strategy | | | |
| --- | --- | --- | --- |
|  | **Early invasive strategy**  **N=996** | **Initial conservative strategy**  **N=355** | **Standardized mean difference** |
| Age, years | 65.8±9.9 | 65.5±10.8 | 0.02 |
| Women | 34.8 | 34.8 | 0.0000 |
| **Cardiovascular risk factors** | | | |
| Hypercholesterolemia | 53.7 | 53.7 | 0.001 |
| Hypertension | 86.7 | 86.0 | 0.02 |
| Current smoking | 28.6 | 28.6 | 0.0006 |
| Family history of CAD | 33.8 | 34.1 | -0.006 |
| **History of ischemic heart disease** | |  |  |
| Chronic coronary syndrome | 31.9 | 30.4 | 0.03 |
| Prior myocardial infarction | 25.4 | 23.9 | 0.03 |
| Prior CABG | 6.0 | 5.6 | 0.01 |
| Prior PCI | 21.6 | 21.1 | 0.01 |
| **History of cardiovascular disease** | |  |  |
| Peripheral artery disease | 4.0 | 3.9 | 0.001 |
| Prior heart failure | 8.2 | 8.6 | -0.01 |
| Prior stroke or TIA | 4.6 | 4.6 | 0.003 |
| **Other comorbidities** |  |  |  |
| Chronic kidney disease | 13.4 | 13.1 | 0.01 |
| **Clinical presentation on hospital admission** | |  |  |
| Heart rate, bpm | 81.8±19.0 | 81.7±17.7 | 0.006 |
| SBP, mmHg | 146.3±25.5 | 145.9±23.7 | 0.01 |
| **Outcomes** |  |  | **P value** |
| PCI complications | 4.8 | 6.0 | 0.36 |
| Risk ratio (95% CI) | 0.78 (0.46 – 1.33) | |  |
| Data are expressed as weighted mean ± standard deviation or weighted rate, unless otherwise specified.  Abbreviations: bpm=beats per minute; CABG=coronary artery bypass graft; CAD= coronary artery disease; PCI=percutaneous coronary intervention; SBP=systolic blood pressure; TIA= Transient ischemic attack. | | | |

| **Table S18.** Inverse probability of treatment weighting: complications in nondiabetic patients undergoing PCI stratified by initial treatment strategy | | | |
| --- | --- | --- | --- |
|  | **Early invasive strategy**  **N=2,463** | **Initial conservative strategy**  **N=875** | **Standardized mean difference** |
| Age, years | 61.9±11.2 | 61.9±11.5 | 0.0000 |
| Women | 25.5 | 25.2 | 0.008 |
| **Cardiovascular risk factors** | | | |
| Hypercholesterolemia | 47.7 | 47.7 | -0.0005 |
| Hypertension | 72.4 | 72.5 | -0.002 |
| Current smoking | 40.9 | 40.8 | 0.002 |
| Family history of CAD | 34.5 | 33.5 | 0.02 |
| **History of ischemic heart disease** | |  |  |
| Chronic coronary syndrome | 25.7 | 25.4 | 0.007 |
| Prior myocardial infarction | 19.9 | 20.3 | -0.01 |
| Prior CABG | 2.5 | 2.5 | -0.003 |
| Prior PCI | 14.9 | 15.3 | -0.01 |
| **History of cardiovascular disease** | |  |  |
| Peripheral artery disease | 3.6 | 3.1 | 0.02 |
| Prior heart failure | 3.6 | 3.8 | -0.007 |
| Prior stroke or TIA | 4.1 | 4.0 | 0.001 |
| **Other comorbidities** |  |  |  |
| Chronic kidney disease | 5.7 | 5.9 | -0.01 |
| **Clinical presentation on hospital admission** | |  |  |
| Heart rate, bpm | 79.3±18.1 | 79.3±19.8 | -0.0009 |
| SBP, mmHg | 144.1±25.8 | 143.9±23.9 | 0.005 |
| **Outcomes** |  |  | **P value** |
| PCI complications | 3.4 | 3.0 | 0.57 |
| Risk ratio (95% CI) | 1.14 (0.73 – 1.78) | |  |
| Data are expressed as weighted mean ± standard deviation or weighted rate, unless otherwise specified.  Abbreviations: bpm=beats per minute; CABG=coronary artery bypass graft; CAD= coronary artery disease; PCI=percutaneous coronary intervention; SBP=systolic blood pressure; TIA= Transient ischemic attack. | | | |

| **Table S19.** Inverse probability of treatment weighting: major bleeding stratified by diabetes status | | | |
| --- | --- | --- | --- |
|  | **Diabetic patients**  **N=2,343** | **Nondiabetic patients**  **N=5,246** | **Standardized mean difference** |
| Age, years | 64.9±10.7 | 64.6±12.1 | 0.02 |
| Women | 32.3 | 32.3 | -0.0007 |
| **Cardiovascular risk factors** | | | |
| Hypercholesterolemia | 48.0 | 48.4 | -0.007 |
| Hypertension | 78.1 | 77.8 | 0.006 |
| Current smoking | 32.6 | 32.7 | -0.002 |
| Family history of CAD | 34.6 | 34.9 | -0.006 |
| **History of ischemic heart disease** | |  |  |
| Chronic coronary syndrome | 30.8 | 30.5 | 0.006 |
| Prior myocardial infarction | 23.0 | 23.3 | -0.006 |
| Prior CABG | 4.4 | 4.3 | 0.003 |
| Prior PCI | 15.6 | 15.5 | 0.0006 |
| **History of cardiovascular disease** | |  |  |
| Peripheral artery disease | 3.9 | 3.7 | 0.007 |
| Prior heart failure | 6.3 | 6.2 | 0.005 |
| Prior stroke or TIA | 4.9 | 5.1 | -0.007 |
| **Other comorbidities** |  |  |  |
| Chronic kidney disease | 8.8 | 8.9 | -0.003 |
| **Clinical presentation on hospital admission** | |  |  |
| Heart rate, bpm | 82.6±18.8 | 82.4±20.9 | 0.01 |
| SBP, mmHg | 142.9±25.9 | 143.3±25.8 | -0.01 |
| **Outcomes** |  |  | **P value** |
| Major bleeding | 2.1 | 1.1 | <0.001 |
| Risk ratio (95% CI) | 1.90 (1.30 – 2.78) | |  |
| Data are expressed as weighted mean ± standard deviation or weighted rate, unless otherwise specified.  Abbreviations: bpm=beats per minute; CABG=coronary artery bypass graft; CAD= coronary artery disease; PCI=percutaneous coronary intervention; SBP=systolic blood pressure; TIA= Transient ischemic attack. | | | |

| **Table S20.** Inverse probability of treatment weighting: major bleeding in diabetic patients stratified by initial treatment strategy | | | |
| --- | --- | --- | --- |
|  | **Early invasive strategy**  **N=1,016** | **Initial conservative strategy**  **N=1,327** | **Standardized mean difference** |
| Age, years | 66.8±9.8 | 66.9±10.6 | -0.006 |
| Women | 38.7 | 38.6 | 0.002 |
| **Cardiovascular risk factors** | | | |
| Hypercholesterolemia | 53.2 | 53.1 | 0.003 |
| Hypertension | 86.9 | 86.7 | 0.005 |
| Current smoking | 24.6 | 24.8 | -0.003 |
| Family history of CAD | 34.1 | 34.1 | 0.0001 |
| **History of ischemic heart disease** | |  |  |
| Chronic coronary syndrome | 33.4 | 33.2 | 0.003 |
| Prior myocardial infarction | 26.4 | 27.2 | -0.01 |
| Prior CABG | 6.3 | 6.4 | -0.005 |
| Prior PCI | 18.8 | 18.6 | 0.004 |
| **History of cardiovascular disease** | |  |  |
| Peripheral artery disease | 4.5 | 4.7 | -0.01 |
| Prior heart failure | 9.3 | 9.2 | 0.003 |
| Prior stroke or TIA | 6.0 | 5.9 | 0.006 |
| **Other comorbidities** |  |  |  |
| Chronic kidney disease | 14.0 | 14.3 | -0.008 |
| **Clinical presentation on hospital admission** | |  |  |
| Heart rate, bpm | 85.3±22.8 | 84.6±19.8 | 0.03 |
| SBP, mmHg | 143.8±26.2 | 143.9±27.0 | -0.002 |
| **Outcomes** |  |  | **P value** |
| Major bleeding | 0.8 | 2.8 | <0.001 |
| Risk ratio (95% CI) | 0.26 (0.12 – 0.57) | |  |
| Data are expressed as weighted mean ± standard deviation or weighted rate, unless otherwise specified.  Abbreviations: bpm=beats per minute; CABG=coronary artery bypass graft; CAD= coronary artery disease; PCI=percutaneous coronary intervention; SBP=systolic blood pressure; TIA= Transient ischemic attack. | | | |

| **Table S21.** Inverse probability of treatment weighting: major bleeding in nondiabetic patients stratified by initial treatment strategy | | | |
| --- | --- | --- | --- |
|  | **Early invasive strategy**  **N=2,497** | **Initial conservative strategy**  **N=2,749** | **Standardized mean difference** |
| Age, years | 63.1±11.2 | 63.2±12.5 | -0.003 |
| Women | 29.5 | 29.5 | -0.0001 |
| **Cardiovascular risk factors** | | | |
| Hypercholesterolemia | 46.2 | 46.3 | -0.0006 |
| Hypertension | 73.7 | 73.7 | -0.0003 |
| Current smoking | 36.8 | 36.7 | 0.0003 |
| Family history of CAD | 35.0 | 34.9 | 0.001 |
| **History of ischemic heart disease** | |  |  |
| Chronic coronary syndrome | 28.5 | 28.8 | -0.006 |
| Prior myocardial infarction | 21.2 | 21.2 | 0.0007 |
| Prior CABG | 3.3 | 3.3 | 0.002 |
| Prior PCI | 14.3 | 14.2 | 0.001 |
| **History of cardiovascular disease** | |  |  |
| Peripheral artery disease | 3.0 | 3.0 | 0.0005 |
| Prior heart failure | 4.7 | 4.7 | -0.0006 |
| Prior stroke or TIA | 4.8 | 4.6 | 0.009 |
| **Other comorbidities** |  |  |  |
| Chronic kidney disease | 6.6 | 6.3 | 0.01 |
| **Clinical presentation on hospital admission** | |  |  |
| Heart rate, bpm | 81.3±20.2 | 81.2±19.7 | 0.004 |
| SBP, mmHg | 142.9±26.1 | 142.9±24.9 | -0.004 |
| **Outcomes** |  |  | **P value** |
| Major bleeding | 1.0 | 1.2 | 0.55 |
| Risk ratio (95% CI) | 0.86 (0.51 – 1.44) | |  |
| Data are expressed as weighted mean ± standard deviation or weighted rate, unless otherwise specified.  Abbreviations: bpm=beats per minute; CABG=coronary artery bypass graft; CAD= coronary artery disease; PCI=percutaneous coronary intervention; SBP=systolic blood pressure; TIA= Transient ischemic attack. | | | |

| **Table S22.** Inverse probability of treatment weighting: clinical factors and outcomes stratified by age subgroup and treatment strategy in diabetic patients | | | | | | |
| --- | --- | --- | --- | --- | --- | --- |
| **Characteristics** | **Diabetic patients** | | | | | |
|  | **Age <65 years** | | | **Age ≥65 years** | | |
|  | **Early invasive strategy**  **(n=489)** | **Initial conservative strategy**  **(n=469)** | **Standardized mean difference** | **Early invasive strategy**  **(n=683)** | **Initial conservative strategy**  **(n=858)** | **Standardized mean difference** |
| Age, years | 56.5±6.2 | 56.5±6.6 | -0.004 | 73.3±5.4 | 73.4±6.3 | -0.01 |
| Women | 28.8 | 29.1 | -0.01 | 44.2 | 44.1 | 0.0002 |
| **Cardiovascular risk factors** | |  |  |  |  |  |
| Hypercholesterolemia | 55.2 | 55.4 | -0.01 | 53.2 | 53.4 | -0.003 |
| Hypertension | 80.8 | 80.5 | 0.01 | 91.2 | 91.2 | -0.001 |
| Current smoking | 39.2 | 39.0 | 0.003 | 15.2 | 15.5 | -0.01 |
| Family history of CAD | 41.4 | 41.3 | 0.003 | 28.7 | 29.0 | -0.01 |
| **History of ischemic heart disease** | |  |  |  |  |  |
| Chronic coronary syndrome | 32.1 | 32.0 | 0.004 | 35.0 | 34.7 | 0.01 |
| Prior myocardial infarction | 25.4 | 25.3 | 0.003 | 27.4 | 28.4 | -0.02 |
| Prior CABG | 5.1 | 5.2 | -0.004 | 7.0 | 7.3 | -0.01 |
| Prior PCI | 17.5 | 17.3 | 0.003 | 18.9 | 18.9 | <0.001 |
| **History of cardiovascular disease** | |  |  |  |  |  |
| Peripheral artery disease | 3.3 | 3.3 | -0.001 | 5.0 | 5.2 | -0.01 |
| Prior heart failure | 7.2 | 7.4 | -0.01 | 10.4 | 10.3 | 0.01 |
| Prior stroke or TIA | 4.5 | 4.5 | 0.003 | 6.9 | 6.6 | 0.01 |
| **Other comorbidities** | |  |  |  |  |  |
| Chronic kidney disease | 7.0 | 7.0 | 0.003 | 17.7 | 18.4 | -0.02 |
| **Clinical presentation on hospital admission** | | |  |  |  |  |
| Mean heart rate (SD), bpm | 84.1±19.6 | 84.0±19.9 | 0.01 | 84.4±21.1 | 84.3±20.0 | 0.002 |
| Mean SBP (SD), mmHg | 146.3±25.7 | 146.2±26.6 | 0.004 | 143.5±26.9 | 143.7±27.0 | -0.01 |
| **Outcomes** | |  | ***P* value** |  |  | ***P* value** |
| 30-day mortality | 1.3 | 2.0 | 0.3796 | 3.7 | 6.6 | 0.0109 |
| Risk ratio (95% CI) | 0.63 (0.23 – 1.76) | | 0.3808 | 0.55 (0.34 – 0.89) | | 0.0147 |
| Data are expressed as weighted means (standard deviation) or weighted percentages, unless otherwise specified.  Abbreviations: bpm, beats per minute; CABG, coronary artery bypass graft; CAD, coronary artery disease; PCI, percutaneous coronary intervention; SBP, systolic blood pressure; TIA, transient ischemic attack. | | | | | | |

| **Table S23.** Inverse probability of treatment weighting: clinical factors and outcomes stratified by age subgroup and treatment strategy in non-diabetic patients | | | | | | |
| --- | --- | --- | --- | --- | --- | --- |
| **Characteristics** | **Nondiabetic patients** | | | | | |
|  | **Age <65 years** | | | **Age ≥65 years** | | |
|  | **Early invasive strategy**  **(n=1674)** | **Initial conservative strategy**  **(n=1439)** | **Standardized mean difference** | **Early invasive strategy**  **(n=1232)** | **Initial conservative strategy**  **(n=1310)** | **Standardized mean difference** |
| Mean age (SD), years | 54.3±7.2 | 54.3±7.4 | 0.0013 | 73.9±6.0 | 74.0±45.16.4 | -0.02 |
| Women | 25.0 | 24.8 | 0.003 | 36.9 | 36.4 | 0.01 |
| **Cardiovascular risk factors** | |  |  |  |  |  |
| Hypercholesterolemia | 47.8 | 47.7 | 0.003 | 45.6 | 45.1 | 0.01 |
| Hypertension | 65.6 | 65.5 | 0.0020 | 82.9 | 82.8 | 0.004 |
| Current smoking | 50.6 | 50.5 | 0.002 | 19.2 | 19.3 | -0.003 |
| Family history of CAD | 37.8 | 37.9 | -0.001 | 30.5 | 30.1 | 0.01 |
| **History of ischemic heart disease** | |  |  |  |  |  |
| Chronic coronary syndrome | 24.3 | 24.6 | -0.01 | 33.5 | 33.8 | -0.01 |
| Prior myocardial infarction | 18.7 | 18.8 | -0.003 | 22.9 | 22.3 | 0.01 |
| Prior CABG | 2.1 | 2.1 | -0.001 | 5.5 | 5.1 | 0.02 |
| Prior PCI | 13.9 | 14.0 | -0.001 | 14.0 | 14.0 | -0.002 |
| **History of cardiovascular disease** | |  |  |  |  |  |
| Peripheral artery disease | 1.5 | 1.5 | 0.01 | 4.6 | 4.6 | 0.0002 |
| Prior heart failure | 2.5 | 2.5 | -0.002 | 7.1 | 7.2 | -0.002 |
| Prior stroke or TIA | 2.9 | 2.8 | 0.01 | 6.4 | 6.2 | 0.01 |
| **Other comorbidities** | |  |  |  |  |  |
| Chronic kidney disease | 3.0 | 2.9 | 0.003 | 9.9 | 9.8 | 0.01 |
| **Clinical presentation on hospital admission** | | |  |  |  |  |
| Mean heart rate (SD), bpm | 80.5±18.3 | 80.5±18.8 | -0.002 | 82.2±21.9 | 82.0±20.9 | 0.01 |
| Mean SBP (SD), mmHg | 144.1±25.5 | 144.0±24.4 | 0.01 | 142.1±26.2 | 142.3±25.8 | -0.01 |
| **Outcomes** | |  | ***P* value** |  |  | ***P* value** |
| 30-day mortality | 1.0 | 1.4 | 0.32 | 2.7 | 4.5 | 0.01 |
| Risk ratio (95% CI) | 0.71 (0.37 – 1.38) | | 0.32 | 0.59 (0.38 – 0.90) | | 0.02 |
| Data are expressed as weighted means (standard deviation) or weighted percentages, unless otherwise specified.  Abbreviations: bpm, beats per minute; CABG, coronary artery bypass graft; CAD, coronary artery disease; PCI, percutaneous coronary intervention; SBP, systolic blood pressure; TIA, transient ischemic attack. | | | | | | |

# **REFERENCES**

1. National Health Interview Survey- Adult Tobacco Use Information. 2017. <https://www.cdc.gov/nchs/nhis/tobacco/tobacco_glossary.htm>.
2. Buuren S, Groothuis-Oudshoorn C. MICE: Multivariate Imputation by Chained Equations in R. Journal of Statistical Software 2011; 45.
3. Austin PC, Stuart EA. Moving towards best practice when using inverse probability of treatment weighting (IPTW) using the propensity score to estimate causal treatment effects in observational studies. Stat Med 2015; 34(28): 3661-79
4. Katz D, Baptista J, Azen SP, et al. Obtaining Confidence Intervals for the Risk Ratio in Cohort Studies. *Biometrics* 1978;34(3):469-74. doi: 10.2307/2530610
5. Dongsheng Y, Dalton JE. A unified approach to measuring the effect size between two groups using SAS®: SAS global forum 2012: statistics and data analysis. SAS Global Forum. 2012: 335-2012. <https://support.sas.com/resources/papers/proceedings12/335-2012.pdf>.
